# Comparison of measles IgG enzyme immunoassays (EIA) versus plaque reduction neutralization test (PRNT) for measuring measles serostatus: a systematic review of head-to-head analyses of measles IgG EIA and PRNT

**DOI:** 10.1186/s12879-023-08199-8

**Published:** 2023-05-31

**Authors:** Chelsea S. Lutz, Alvira Z. Hasan, Shelly Bolotin, Natasha S. Crowcroft, Felicity T. Cutts, Eugene Joh, Stacie Loisate, William J. Moss, Selma Osman, Kyla Hayford

**Affiliations:** 1 Department of International Health, Johns Hopkins University Bloomberg School of Public Health, Baltimore, MD USA; 2 International Vaccine Access Center, Johns Hopkins University Bloomberg School of Public Health, Baltimore, MD USA; 3 Centre for Vaccine Preventable Diseases, Dalla Lana School of Public Health, University of Toronto, Toronto, ON Canada; 4 Public Health Ontario, Toronto, ON Canada; 5 Department of Laboratory Medicine and Pathobiology, University of Toronto, Toronto, Canada; 6 Department of Immunisation, Vaccines and Biologicals, World Health Organization, Geneva, Switzerland; 7 Department of Infectious Disease Epidemiology, London School of Hygiene and Tropical Medicine, University of London, London, UK; 8 Department of Epidemiology, Johns Hopkins University Bloomberg School of Public Health, Baltimore, MD USA; 9 Pfizer Vaccines, Inc., New York City, NY USA

**Keywords:** Diagnostic accuracy, EIA, ELISA, IgG, Measles, Measles IgG serology, PRN, PRNT, Sensitivity, Serology, Serosurveillance, Specificity

## Abstract

**Background:**

As countries move towards or achieve measles elimination status, serosurveillance is an important public health tool. However, a major challenge of serosurveillance is finding a feasible, accurate, cost-effective, and high throughput assay to measure measles antibody concentrations and estimate susceptibility in a population. We conducted a systematic review to assess, characterize, and – to the extent possible – quantify the performance of measles IgG enzyme-linked assays (EIAs) compared to the gold standard, plaque reduction neutralization tests (PRNT).

**Methods:**

We followed the PRISMA statement for a systematic literature search and methods for conducting and reporting systematic reviews and meta-analyses recommended by the Cochrane Screening and Diagnostic Tests Methods Group. We identified studies through PubMed and Embase electronic databases and included serologic studies detecting measles virus IgG antibodies among participants of any age from the same source population that reported an index (any EIA or multiple bead-based assays, MBA) and reference test (PRNT) using sera, whole blood, or plasma. Measures of diagnostic accuracy with 95% confidence intervals (CI) were abstracted for each study result, where reported.

**Results:**

We identified 550 unique publications and identified 36 eligible studies for analysis. We classified studies as high, medium, or low quality; results from high quality studies are reported. Because most high quality studies used the Siemens Enzygnost EIA kit, we generate individual and pooled diagnostic accuracy estimates for this assay separately. Median sensitivity of the Enzygnost EIA was 92.1% [IQR = 82.3, 95.7]; median specificity was 96.9 [93.0, 100.0]. Pooled sensitivity and specificity from studies using the Enzygnost kit were 91.6 (95%CI: 80.7,96.6) and 96.0 (95%CI: 90.9,98.3), respectively. The sensitivity of all other EIA kits across high quality studies ranged from 0% to 98.9% with median (IQR) = 90.6 [86.6, 95.2]; specificity ranged from 58.8% to 100.0% with median (IQR) = 100.0 [88.7, 100.0].

**Conclusions:**

Evidence on the diagnostic accuracy of currently available measles IgG EIAs is variable, insufficient, and may not be fit for purpose for serosurveillance goals. Additional studies evaluating the diagnostic accuracy of measles EIAs, including MBAs, should be conducted among diverse populations and settings (e.g., vaccination status, elimination/endemic status, age groups).

**Supplementary Information:**

The online version contains supplementary material available at 10.1186/s12879-023-08199-8.

## Introduction

Measles is a highly infectious, acute systemic viral infection, estimated to cause over 100,000 deaths annually, despite widespread use of a safe and effective vaccine [1]. Between 2000 and 2020, an estimated 31.7 million deaths were averted because of measles vaccination and estimated global measles deaths declined by 94% [2]. In 2020, global coverage of the first dose of measles containing vaccine (MCV1) was estimated at 84% [3]. Coverage of a second measles-containing dose (MCV2) has accelerated in the last decade: as of 2020, 179 countries introduced MCV2 and global coverage was 70% [3, 4]. However, this level of coverage is inadequate to control measles, and progress has been stymied by persistent gaps in measles vaccination coverage, with wide variations within and across populations. Global cases resurged since 2016, with lapses in coverage contributing to high numbers of cases and deaths in 2018 and 2019 [5, 6]. In 2019, there were almost 870,000 cases and over 200,000 deaths – the greatest number of cases since 1996 [7, 8]. Since the COVID-19 pandemic, the measles vaccination coverage has declined and, as of 2021, 40 million children have missed a measles vaccine dose [9].

High quality vaccination programs routinely rely on two sources of data to identify measles outbreaks and populations at highest risk: 1) vaccination coverage monitoring; and 2) measles case surveillance. However, many countries lack high-quality vaccine coverage and/or disease incidence data. Serosurveillance for immunoglobulin G (IgG) antibodies to measles virus can account for waning vaccine-induced immunity, inaccurate recordkeeping, and immunity from natural infection, and is therefore potentially a more direct tool to identify susceptible populations and intervene prior to an outbreak [10]. Between 1996 and 2004, 17 European countries and Australia used serosurveillance to classify progress towards elimination status, including gaps in coverage and risk of localized outbreaks and epidemics [11]. In principle, serosurveillance, which allows the assessment of vaccine failure as well as infection, can also be used to assess the impact of vaccination programs, vaccine effectiveness, transmission dynamics, and predict risk of future epidemics [12].

A challenge of serosurveillance is finding a feasible, accurate, and high throughput assay to measure measles antibody level and estimate susceptibility in a population. The plaque reduction neutralization test (PRNT) is a functional antibody assay that measures the neutralization activity of measles antibodies regardless of isotype. A neutralizing antibody (NAb) is an antibody that defends a cell from a pathogen or infectious particle by neutralizing any effect it has biologically. Neutralization renders the particle no longer infectious or pathogenic [13]. Neutralization assays are considered the “gold standard” for determining protective immunity [12, 14–16]. A threshold of measles neutralizing antibody levels of 120 mIU/mL is often considered the correlate of protection although other thresholds, such as 200 mIU/mL, are used depending on which international reference sera was used to calibrate the assay and the objective of the test [17–19]. Quantitative values from PRNT show good correlation with immune status and predict protection against infection and disease [20]. However, using PRNT in large serological studies is impractical because it is technically demanding, expensive, conducted in a limited number of laboratories around the world, labor-intensive, time-consuming, and the procedures and interpretation of PRNTs are difficult to standardize between laboratories [20, 21]. Enzyme immunosorbent assays (EIA) are rapid, relatively inexpensive, higher throughput assays that can be performed in most laboratories with basic equipment using commercially available assays [22]. However, EIAs are not functional assays and measure IgG isotype-specific epitopes regardless of neutralization capacity [23]. Multiple studies have reported that EIA results are less sensitive than PRNT, especially in the context of low antibody levels [14, 21, 24–27]. This may lead to individuals being misclassified by EIA as susceptible to measles in populations with low antibody levels from vaccination as a result of immunological immaturity, interference by passively acquired maternal antibodies, or with waning antibody levels after prolonged periods since vaccination, especially in the absence of boosting from exposure to wild-type virus [22]. Uniquely for measles, minor reductions in EIA sensitivity can have substantial consequences for estimating population immunity due to its high herd immunity threshold, which could result in a misallocation of resources to increase vaccination coverage.

As the use of serosurveillance to evaluate population susceptibility to and seroprotection against measles increases, understanding the diagnostic accuracy of EIAs compared to the gold standard is critical to select an appropriate assay for the target population that can achieve the research or programmatic goals [28, 29]. Although direct comparisons of measles IgG EIA results with PRNTs have been periodically reported in the literature, such comparisons are often not the main objective of the analyses [30] and lack sufficient information about assays and procedures to assess the EIA validity. This systematic review was conducted to assess, characterize, and – to the extent possible – quantify the performance of measles IgG EIAs compared to PRNT.

## Methods

We followed the PRISMA statement for a systematic literature search (Supplementary Table 1) [31, 32] and followed methods for conducting and reporting systematic reviews and meta-analyses recommended by the Cochrane Screening and Diagnostic Tests Methods Group (SDTM) [33].

### Registration and protocol

We documented methods of the analysis and inclusion criteria in a protocol registered with PROSPERO (registration ID: CRD42020170464).

### Eligibility criteria

We included serologic studies with participants of any age from the same source population that reported an index and reference test of measles antibodies using sera, whole blood, or plasma. The index test was any EIA (in-house or commercial, including single or multiple bead-based assays [MBA]) detecting measles virus IgG antibodies. The reference test for the primary analysis was PRNT. Studies that included neutralizing tests (NT) only as the reference test were included in the review but excluded from the primary analysis.

### Information sources

We identified studies through PubMed and Embase electronic databases. The original search was conducted on 28 January 2020 and updated twice on 8 June 2020 and 25 August 2021. After full text screening, we attempted to acquire missing information on results from the primary investigator of studies of potential relevance.

### Search strategy and selection criteria

The search strategies used terms such as “measles”, “measles vaccine”, “enzyme immunoassay”, “EIA”, “viral plaque assay”, and “PRNT”. Full PubMed and Embase search strategies are detailed in supplemental materials, S2. We included studies if the subjects were human, measured measles IgG antibodies using both an EIA and PRNT, and were published from 1946 to the most recent search (25 August 2021). The literature search was not limited by language and non-English studies were included if an English translation could be obtained. We excluded duplicate studies, basic science literature (e.g., vaccine development), conference abstracts, studies with no abstracts, reviews, and meta-analyses. In addition, we conducted snowball search strategies to identify relevant studies that may have been missed by our database searches, including reviewing the reference lists of included studies.

### Study selection

We used Covidence Review Software [34] to maintain search results and conduct all screening processes. Two investigators independently assessed titles and abstracts for eligibility based on the PICOS criteria (Population = participants with and without previous measles infection from all settings, tested for measles virus IgG; Index test = EIA; Comparator = PRNT; Outcomes = EIA vs. PRNT performance, measured by sensitivity, specificity, positive predictive value, negative predictive value, c-statistic, R^2^, kappa, and/or percent agreement; Study design = immunologic studies). Two investigators then screened full-text studies for inclusion using the same criteria. We analyzed outcomes from the remaining relevant research studies. Disagreements between reviewers at all stages were resolved by consensus or involving a third investigator when consensus could not be reached.

### Data abstraction

We developed a data abstraction tool using the Standards for Reporting of Diagnostic Accuracy Studies (STARD) 2015 guidelines [35] and guidance from similarly-focused reviews [36]. We pilot tested the abstraction tool on studies representative of different study designs and data quality and refined it accordingly. All authors commented on the abstraction tool and approved the final version. Four investigators abstracted data from included studies.

We abstracted the following information from each study: 1) study design and setting, e.g., country in which the study was conducted, age of the population, specimen type; 2) EIA results including qualitative and quantitative IgG antibody results, assay type (in-house, commercial); 3) PRNT results including qualitative result, antibody levels, methods for conversion to international units; 4) EIA performance compared to PRN, e.g., sensitivity, specificity, positive predictive value, negative predictive value. We used thresholds as reported in the papers. Each comparison from papers reporting more than one EIA vs. PRNT comparison (eg., multiple EIA or PRNT thresholds, multiple EIA kits, multiple age groups or populations etc.) was reported as separate results. After the data were abstracted, measles elimination status at the time of the study and time since elimination in elimination settings was determined using peer-reviewed and grey literature, based on country and year of specimen collection (or publication year if date of specimen collection was not reported). Elimination status included endemic (the existence of continuous indigenous or imported measles virus transmission that persists for ≥ 12 months in any defined geographical area), interruption (absence of endemic measles virus transmission in a defined geographical area for < 12 months), or elimination (the interruption of endemic measles transmission in a defined geographical area for ≥ 12 months in the presence of a well-performing surveillance system).

### Assessment of methodological quality and data quality classifications

We classified studies as high, medium, or low quality in terms of the metrics reported and the reproducibility of study findings (Table 1).

**Table 1:** Data quality classification definitions for publication abstracted and included in analysis

Classifications	Definition
High quality	Sensitivity and specificity of the EIA kit(s) used reported and two-by-two tables replicating these results generated
Medium quality	Sensitivity and specificity of the EIA kit(s) used directly reported but not enough additional information provided in the text to generate two-by-two tables and replicate results, or Sensitivity and specificity of the EIA kit(s) used not reported but sufficient information provided in the text to create two-by-two tables to estimate the sensitivity and specificity
Low quality	Some measure of correlation or agreement reported, but sensitivity and specificity not reported and not enough information included in the text to estimate them

Medium and low quality studies are described in Supplemental Tables 2A and 2B, but are not included in the main analysis. Papers were excluded from analysis if they did not report data relevant to the study objectives or did not classify the quality of these data. We also assessed the risk of bias for individual studies using a modified version of the Quality Assessment of Diagnostic Accuracy Studies (QUADAS-2) revised tool for Cochrane reviews [37].

**Table 2:** Descriptive characteristics of studies evaluating EIA assays compared to PRNT (high quality)

Study	Country of sample collection	Elimination status at time of study	Objective	N of samples tested	Age(s)	Type of study subjects/samples	Eligibility criteria	EIA kit	EIA threshold	PRNT threshold	Subsample selected
**Cohen 2006 ** [25]	United Kingdom	Endemic	Diagnostic accuracy	100	NR	Serum samples submitted for immunity testing	Random sample or source unrelated to exposure or outcome	Siemens Enzygnost; Microimmun	< 0.1 O.D; < 1.1 O.D	Batch specific cut off ^c^	All tested
**Cohen 2008** [21]	Kenya	Endemic	Diagnostic accuracy	210	9 m	Residual serum samples from separate study collected 4 weeks post measles vaccination	Random sample or source unrelated to exposure or outcome	Siemens Enzygnost	< 0.1 O.D. (Automatic, Manual)	≥ 120 mIU/mL	All negative, low positive and unusual PRN profiles, and random subset of high PRN. Positives selected for EIA
**Coughlin 2021** [19]	USA, Tajikistan	Eliminated (US)^e^; Endemic (Tajikistan)	Diagnostic accuracy	140; 212; 516	6 m—adults	Residual serum samples obtained from routine case-based surveillance (US), early revaccination cohort (US) and a serosurvey (Tajikistan)	Random sample or source unrelated to exposure or outcome	In house MBA	MeV N(< 9.5mIU/mL); MeV WVA_L_ (< 137 mIU/mL); MeV WVA_c_ (< 153mIU/mL)	≥ 120 mIU/mL^b^	All tested
**deSouza 1991** [44]	Brazil	Endemic	Diagnostic accuracy	181	< 18yrs	Serum samples obtained from measles vaccinated children and umbilical cord	Results from previous tests	In house EIA	DOD reading: ≤ 0.12	NR	All tested
**Dorigo-Zetsma 2015** [45]	The Netherlands	Eliminated	Diagnostic accuracy	154	≥ 18yrs	HCWs born after 1960 working at departments with reported measles cases	Recruited from community or healthcare setting, not related to measles infection or vaccination	Diasorin; Siemens Enzygnost; Vidas; In house MBA	< 13.5 AU/ml; < 0.1 O.D.; < 0.5 Test values; < 120 mIU/mL	≥ 120 mIU/mL	All tested
**Fowlkes 2011** [47]	Malawi	Endemic	Persistence of vaccine-induced measles antibody	2344	6- 36 m & mothers (ages NR)	Samples collected from children at 6,9,12, 20, 24 and for some, 30–36 months. Subset of mothers HIV infected and children HIV infected or exposed	Recruited from community or healthcare setting, not related to measles infection or vaccination	Trinity Biotech	NR	≥ 120 mIU/mL	Random EIA subset tested on PRN
**Goncalves 1999** [48]	Portugal	Endemic	Diagnostic accuracy	43	11-14 m	Serum samples obtained from children 11-14 m who were at the age of routine measles vaccination	Children at the age of routine measles vaccination	Diagnostica, Merck	< 40 mIU/mL; < 100 mIU/mL	≥ 40 mIU/mL; 100 mIU/mL	All tested
**Hatchette 2017** [26]	Canada	Eliminated^e^	Diagnostic accuracy	148	NR	Residual samples submitted for immunity testing previously categorized as immune (n = 50), nonimmune (*n* = 50), or equivocal (*n* = 48) by EIA	Results from previous tests	BioPlex 2200 MMRV IgG	< 0.13 AU/mL ^a^	> 192 mIU/mL^b^	All tested
**Lee 1999** [54]	United Kingdom	Endemic	Diagnostic accuracy	85	NR	Serum samples of with known range of PRNT titers (< 200mIU/mL to > 4000 mIU/mL) selected	Results from previous tests	In house EIA	< 200 mIU/mL	> 200 mIU/mL^b^	Random subset of negative, low positive, medium positive and high positive PRN titers selected for EIA
**Mao 2009** [56]	China	Endemic	Diagnostic accuracy	52; 47	NR	Serum samples selected based on measles antibody titers (< 1:4, 1:4, 1:120, < 1:1052) obtained from 5 provinces	Results from previous tests	German Virion/Serion; IBL	< 150 mIU/mL; < 8 U (unit) / ml	≥ 1: 4^b^	All tested
**Ratnam 1995** [63]	Canada	Endemic	Diagnostic accuracy	1287, 229; 229	12-15 m, 1-16yrs; 1-16yrs	Age 12-15 m: Serum samples pre and post measles vaccination; Age 1-16y: Serum samples from children with prior MMR vaccination with PRN titers 8 to 10,000	Selected based on vaccination status	Siemens Enzygnost/Dade Behring; Diamedix; Measlestat; Vidas	< 0.1 O.D.; < 15 EIA unit; ≤ 0.79 Predicted value index; < 0.5 Test value threshold	≥ 120 mIU/mL, 8mIU/mL	All tested
Tischer **Andrews 2007** [27]	United Kingdom	Endemic	Diagnostic accuracy	151	NR	Serum samples selected by antibody concentration (high positives, low positives, equivocals and negatives	Panel of samples with known antibody concentrations tested	Siemens Enzygnost	< 0.1 O.D	≥ 40 ± 20 mIU/mL^b^	All tested
**Warrener 2018** [67]	Uganda	Endemic	Population-based seroprevalence study	113; 203	4-15 m & 12-75 m	4-15 m: Children from health clinic with no record of measles vaccination 12-75 m: Children from outpatient department, majority received measles vaccine by recall	Recruited from community or healthcare setting, not related to measles infection or vaccination	Siemens Enzygnost	NR	≥ 120 mIU/mL	All tested

*DOD* Difference between means, *EIA* Enzyme immunoassay, *HCW* Healthcare worker, *MBA* Multiplex bead assay, *MMR* Measles, mumps and rubella vaccine, *MeV N*, Recombinant measles virus nucleoprotein, *MeV WVAL* Laboratory-produced purified measles whole-virus antigen, *MeV WVA*_*c*_ Commercially produced whole-virus antigen, *MIA* Multiplex immunoassay, *mIU/mL* milli-international units per milliliter, *m* Month, *NA* Not available, *NR* Not reported, *O.D* Optical density, *PRNT* Plaque reduction neutralization test, *USA* United States of America, *yrs* years

^a^EIA thresholds reported did not use or did not explicitly report to use manufacturers recommendation

^b^Reported to use PRNT methods other than those described in Albrecht et. al. 1981 or did not describe methodology

^c^mIU/mL calculated separately for each batch based on 2nd measles International Standard. Threshold based on adjusted 1:8 dilution

^e^Elimination status was assumed using publication year as date of specimen collection was not reported

### Data analysis

Measures of diagnostic accuracy with 95% confidence intervals (CI) were abstracted for each study result, where reported. Data were also abstracted to generate the four cell values of a two-by-two table, where available, and used to recalculate the sensitivity, specificity, positive predictive value (PPV), and negative predictive value (NPV) with 95% CIs for each index-reference test comparison. Recalculated metrics were used in the main analysis. If recalculated metrics were not available (e.g., medium quality studies in the supplementary materials), the reported measures were used to calculate metrics. Indeterminate or equivocal EIA results were handled in the same way as reported by authors in the study (i.e., excluded or treated as positives or negative) in the primary analysis. If the data reported by authors or methods for treating equivocals were unclear, authors were contacted for additional information or to verify calculations. If no information could be obtained from the authors, investigators came to a consensus regarding whether to include the study (*n* = 1). We conducted sensitivity analysis by reclassifying equivocal or indeterminant EIA results (e.g., treating as negative, as positive, or excluding from analysis).

Diagnostic accuracy measures were presented for high (main text) and medium (supplementary materials) quality studies only. Differences between studies was assessed by visual examination of forest plots using Stata/IC (version 16.1) [38]. The diagnostic accuracy measures for high quality comparisons that used the Enzynost kits were also presented in a hierarchical summary receiver operating characteristic (HSROC) curve, indicating pooled sensitivity and specificity with 95% confidence regions around the summary estimates. This was used to explain observed differences in accuracy between EIA kits.

We generated a QUADAS figure for all studies using R (version 3.6.1) (Supplementary Fig. 1). For studies with multiple groups (e.g., multiple age groups or multiple EIA kits), we reassigned QUADAS-2 assessments so that a single result was presented per domain for each study. This was done by following an algorithm that compared multiple results within each QUADAS-2 domain and assigned the worst rating as the final, overall assessment per study.

## Results

### Search results

**Fig. 1:**
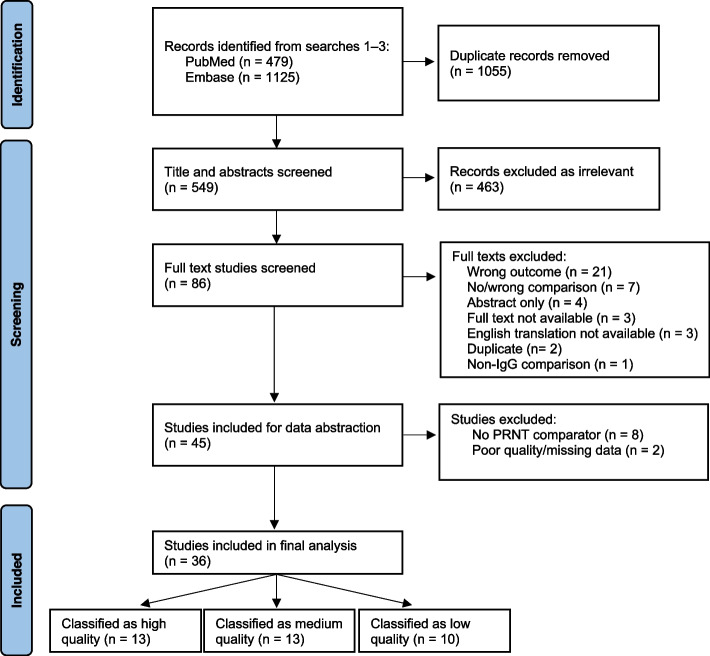
PRISMA flow diagram for database searches and study inclusion, Studies evaluating EIA assays compared to PRNT (high quality)

A total of 549 results were identified through the literature searches after removing duplicates, of which 463 studies were excluded at title and abstract screening, and 41 were excluded at full-text review (Fig. 1). Of the 45 studies included for abstraction, ten were excluded after detailed assessment because a PRNT or comparable test was not used (*n* = 8) or relevant results were not reported (*n* = 2). One additional study was included through a snowball search. Thirty-six studies were included for review and 26 for analysis [19, 21, 25–27, 30, 39–68]. Thirteen were classified as high quality, 13 as medium quality, and 10 as low quality.

### Characteristics of reviewed studies

For the following sections, characteristics described are not mutually exclusive (i.e., studies may have used more than one age group, specimen source, or EIA kit).

#### Study populations

Nine of thirteen high quality studies were conducted in high- or upper-middle-income countries (Brazil, Canada, China, England, Portugal, The Netherlands, United States, and United Kingdom), three in lower-middle- or low-income countries (Kenya, Malawi, and Uganda; Table 2), and one study analyzed specimens from both high and lower-middle income countries (United States, Tajikistan, and Bangladesh). Ten of 13 high quality studies used data from measles endemic settings, two from measles elimination settings, and one from a mix of endemic and elimination settings. Nearly all medium and low quality papers were conducted in high- or upper-middle-income countries.

The number of specimens ranged substantially from 43 to 2344 specimens per study (Table 2). Across all high quality studies, one study used specimens from adults, five from children (< 18 years), two from a mix of adults and children, and five did not report the age range. Five of the seven studies with pediatric specimens included children younger than 12 months of age. The original purpose of the analysis varied by study (e.g., diagnostic accuracy evaluation, serosurveillance, Table 2).

#### Types of EIA kits used

In high quality studies, ten commercial EIAs and two in-house EIAs were compared to PRNT (Table 3). Siemens Enzygnost/Dade Behring (“Enzygnost”) EIA was used most often (*n* = 14 results in 6 studies), followed by the VIDAS® (bioMerieux; “VIDAS”) assay (*n* = 3), and other in-house EIAs (*n* = 2) (Tables 3 and 4). MBAs were used in three studies: one commercial MBA and two studies using in-house MBAs. Medium- and low-quality studies used a wider variety of commercial EIAs as well as in-house EIAs and MBAs (Supplementary Table 2A and 2B).

**Table 3:** High quality studies diagnostic accuracy measures (Siemens Enzygnost/Dade Behring only)

**Study**	N of samples tested	Age	EIA threshold	PRNT threshold	EIA equivocal grouped as	TP	FP	FN	TN	Sensitivity % (95% CI)	Specificity % (95% CI)	PPV % (95% CI)	NPV % (95% CI)	Kappa statistic	R
**Cohen 2006** [25]	100	NR	< 0.1 O.D	Batch specific cut off^a^	Positive	69	0	8	23	89.6 (80.6–95.4)	100.0 (85.2–100.0)	100.0(94.8–100.0)^c^	74.2 (55.4–88.1)^c^	NR	83.0%
**Cohen 2008 **[21]^e^	210	9 m	< 0.1 O.D (Automatic)	≥ 120 mIU/mL	Positive	118	2	60	30	66.3 (58.8–73.2)^d^	93.8 (79.2–99.2)^d^	98.3 (94.1–99.8)^d^	33.3 (23.7–44.1)^d^	NR	0.67^e^
			< 0.1 O.D (Automatic)	≥ 120 mIU/mL	Negative	57	0	121	32	32.0 (25.0–39.0)^d^	100.0 (89.0–100.0)^d^	100.0 (93.7–100.0)^d^	20.9 (14.8–28.2)^d^	NR	0.67^e^
			< 0.1 O.D (Manual)	≥ 120 mIU/mL	Positive	156	10	22	22	87.6 (81.9–92.1)^d^	68.8(50.0–83.9)^d^	94.0 (89.2–97.1)^d^	50.0 (34.6–65.4)^d^	NR	NR
			< 0.1 O.D (Manual)	≥ 120 mIU/mL	Negative	110	1	68	31	62.0 (54.0–69.0)^d^	97.0 (84.0–100.0)^d^	99.1 (95.1–100.0)^d^	31.3 (22.4–41.4)^d^	NR	NR
**Dorigo-Zetsma 2015** [45]	154	≥ 18yrs	< 0.1 O.D	≥ 120 mIU/mL	Positive	139	0	14	1	90.8 (85.1–94.9)	100.0 (2.5–100.0)^c^	100.0 (97.4–100.0)^c^	6.7 (0.2–31.9)^c^	NR	NR
**Ratnam 1995** [63]	1287	12-15 m	< 0.1 O.D	≥ 120 mIU/mL	Excluded	160	4	0	39	100.0 (97.7–100.0)	90.7 (77.9–97.4)	97.6 (93.9–99.3)	100.0 (91.0–100.0)	NR	NR
				≥ 8 mIU/mL	Excluded	623	6	100	538	86.2 (83.4–88.6)	98.9 (97.6–99.6)	99.0 (97.9–99.6)	84.3 (81.3–87.1)	NR	NR
**Ratnam 1995** [63]	229	1-16yrs	< 0.1 O.D	≥ 120 mIU/mL	Excluded	588	41	7	631	98.8 (97.6–99.5)	93.9 (91.8–95.6)	93.5 (91.3–95.3)	98.9 (97.8–99.6)	NR	NR
				≥ 8 mIU/mL	Excluded	164	0	22	17	88.2 (82.6–92.4)	100.0 (80.5–100.0)	100.0 (97.8–100.0)	43.6 (27.8–60.4)	NR	NR
Tischer **2007** [27]	151	NR	< 0.1 O.D	≥ 40 ± 20 mIU/mL^b^	Positive	122	0	6	23	95.3 (90.1–98.3)	100.0 (85.2–100.0)	100.0 (97.0–100.0)^c^	79.3 (60.3–92.0)^c^	NR	NR
**Warrener 2018** [67]	316	4-15 m & 12-75 m	NR	≥ 120 mIU/mL	Positive	183	4	13	116	93.4 (88.9–96.4)	96.7 (91.7–99.1)	97.9 (94.6–99.4)	89.9(83.4–94.5)	NR	0.83
	113	4-15 m	NR	≥ 120 mIU/mL	Positive	122	1	5	95	96.1 (91.1–98.7)	99.0 (94.3–100.0)	99 .2 (95.6–100.0)^c^	95.0 (88.7–98.4)^c^	NR	NR
	203	12-75 m	NR	≥ 120 mIU/mL	Positive	171	3	8	21	95.5 (91.4–98.1)	87.5 (67.6–97.3)	98.3 (95.0–99.6)^c^	72.4 (52.8–87.3)^c^	NR	NR

*CI* Confidence interval, *EIA* Enzyme immunoassay, *FN* False negatives, *FP* False positives, *TN* True negatives, *m* Months, *NPV* Negative predictive value, *NE* Not estimable, *NR* Not reported, *O.D* Optical density, *PRNT* Plaque reduction neutralization test, *PPV* Positive predictive value, *R* Correlation coefficient, *TP* True positives, *yrs* Years

^a^EIA thresholds reported did not use or did not explicitly report to use manufacturers recommendation

^b^Reported to use methods other than those described in Albrecht et. al. 1981 or did not describe methodology

^c^Estimates presented were not reported by authors but calculated using data reported

^d^Cohen 2008 authors reported weighted estimates, unweighted estimates displayed

^e^The authors reported overall correlation for the automated ELISA (0.67) and manual ELISAs (0.59)

^e^All negative, low positive, unusual PRN profiles, and random subset of high PRN positives selected for EIA testing

**Table 4:** High quality studies diagnostic accuracy measures (non-Siemens Enzygnost/Dade Behring only)

Study	N of samples tested	Age	EIA kit	EIA threshold	PRNT threshold	EIA equivocal grouped as	TP	FP	FN	TN	Sensitivity % (95%CI)	Specificity % (95%CI)	PPV %(95%CI)	NPV %(95%CI)	Kappa statistic	R
**Goncalves 1999 **[48]	43	11-14 m	Diagnostica Merck	< 40 mIU/mL	≥ 40 mIU/mL	NR	0	1	4	38	0.0(0.0–60.2)^c^	97.4 (86.5–99.9)	0.0 (0.0–97.5)^c^	90 .5(77.4–97.3)^c^	NR	NR
				< 100 mIU/mL	≥ 100 mIU/mL	NR	0	0	1	42	0.0(0.0–97.5)^c^	100.0 (91.6–100.0)^c^	NE	97.7 (87.7–99.9)^c^	NR	NR
**Ratnam 1995** [63]	229	1-16yrs	Diamedix	< 15 EIA unit	≥ 120 mIU/mL	Excluded	165	21	3	30	98.2 (94.9–99.6)	58.8 (44.2–72.4)	88.7 (83.3–92.9)	90.9 (75.7–98.1)	NR	NR
					≥ 8 mIU/mL	Excluded	185	1	18	15	91.1 (86.3–94.7)	93.8 (69.8–100.0)	99.5 (97.0–100.0)	45.5 (28.1–63.6)	NR	NR
**Dorigo-Zetsma 2015** [45]	154	≥ 18yrs	Diasorin	< 13.5 AU/ml	≥ 120 mIU/mL	Grouped with positives	136	0	17	1	88.9 (82.8–93.4)	100.0 (2.5–100.0)^c^	100.0 (97.3–100.0)^c^	5.6 (0.1–27.3)^c^	NR	NR
**Mao 2009 **[56]	52	NR	German Virion/Serion	< 150 mIU/mL	≥ 1: 4^b^	NR	37	0	2	13	94.9 (82.7–99.4)	100.0 (75.3–100.0)	100.0 (90.5–100.0)	86.7 (59.5–98.3)	0.90	0.88
	47	NR	IBL kit	< 8 U (unit) / ml	≥ 1: 4^b^	NR	20	0	14	13	58.8 (40.7–75.4)	100.0 (75.3–100.0)	100.0 (83.2–100.0)	48.1 (28.7–68.1)	0.44	0.85
**Ratnam 1995** [63]	229	1-16yrs	Measlestat	≤ 0.79 Predicted value index	≥ 120 mIU/mL	Excluded	132	2	22	53	85.7 (79.2–90.8)	96.4 (87.5–99.6)	98.5 (94.7–99.8)	70.7 (59.0–80.6)	NR	NR
					≥ 8 mIU/mL	Excluded	134	0	58	17	69.8 (62.8–76.2)	100.0 (80.5–100.0)	100.0 (97.3–100.0)	22.7 (13.8–33.8)	NR	NR
**Cohen 2006 **[25]	100	NR	Microimmun	< 1.1 O.D	Batch specific cut off^a^	Grouped with positives	69	0	8	23	89.6 (80.6–95.4)	100.0 (85.2–100.0)	100.0 (94.8–100.0)^c^	74.2 (55.4–88.1)^c^	NR	72.0%
**Fowlkes 2011 **[47]^**e**^	2344	6- 36 m; mothers (age NR)	Trinity	NR	≥ 120 mIU/mL	NR	1412	70	135	727	91.3 (89.8–92.6)	91.2 (89.0–93.1)	95.3 (94.1–96.3)^c^	84.3 (81.7–86.7)^c^	NR	NR
**Ratnam 1995** [63]	229	1-16yrs	Vidas	< 0.5 Test value threshold	≥ 120 mIU/mL	Excluded	144	3	15	52	90.6 (84.9–94.6)	94.5 (84.9–98.9)	98.0 (94.2–99.6)	77.6 (65.8–86.9)	NR	NR
					≥ 8 mIU/mL	Excluded	147	0	50	17	74.6 (67.9–80.5)	100.0 (80.5–100.0)	100.0 (97.5–100.0)	25.4 (15.5–37.5)	NR	NR
**Dorigo-Zetsma 2015** [45]	154	≥ 18yrs	Vidas	< 0.5 Test values (TV)	≥ 120 mIU/mL	Grouped with positives	137	0	16	1	89.5 (83.6–93.9)	100.0 (2.5–100.0)^c^	100.0 (97.3–100.0)^c^	5.9 (0.1–28.7)^c^	NR	NR
**Hatchette 2017 **[26]	148	NR	BioPlex 2200	< 0.13 AU/mL	> 192 mIU/mL^b^	Grouped with negatives	55	0	30	63	64.7 (53.6–74.8)	100.0 (94.3–100.0)	100.0 (93.5–100.0)^c^	67.7 (57.3–77.1)^c^	NR	0.64
**Dorigo-Zetsma 2015** [45]	154	≥ 18yrs	In house MBA (MIA)	< 120 mIU/mL	≥ 120 mIU/mL	Grouped with positives	149	0	4	1	97.4 (93.4–99.3)^c^	100.0 (2.5–100.0)^c^	100.0 (97.6–100.0)^c^	20.0 (0.5–71.6)^c^	NR	NR
**Coughlin 2021 **[19]	140	6 m—adults	In house MBA (MeV N)	< 9.5 mIU/mL	≥ 120 mIU/mL^b^	NR	84	9	12	35	87.5 (79.2–93.4)	79.5 (64.7–90.2)	90.3 (82.4–95.5)	74.5 (59.7–86.1)	NR	0.431
			In house MBA (MeV WVA_L_)	< 137 mIU/mL	≥ 120 mIU/mL^b^	NR	91	11	5	33	94.8 (88.3–98.3)	75.0 (59.7–86.8)	89.2 (81.5–94.5)	86.8 (71.9–95.6)	NR	0.827
**Coughlin 2021 **[19]	212	6 m—adults	In house MBA (MeV WVA_L_)	< 137 mIU/mL	≥ 120 mIU/mL^b^	NR	119	12	6	75	95.2 (89.8–98.2)	86.2 (77.1–92.7)	90.8 (84.5–95.2)	92.6 (84.6–97.2)	NR	0.768
			In house MBA (MeV WVA_C_)	< 153 mIU/mL	≥ 120 mIU/mL^b^	NR	119	16	6	71	95.2 (89.8–98.2)	81.6 (71.9–89.1)	88.1 (81.5–93.1)	92.2 (83.8–97.1)	NR	0.716
**deSouza 1991 **[44]	181	< 18 yrs	In house EIA	DOD reading: ≤ 0.12	NR	NR	177	0	2	2	98.9 (96.0–99.9)	100.0 (15.8–100.0)^c^	100.0 (97.9–100.0)^c^	50 .0 (6.8–93.2)^c^	NR	0.81
**Lee 1999 **[54]^**f**^	85	NR	In house EIA	< 200 mIU/mL	> 200 mIU/mL^b^	NR	68	0	1	16	98.6 (92.2–100.0)	100.0 (79.4–100.0)	100.0 (94.7–100.0)	94.1 (71.3–99.9)	0.98	NR

*CI* Confidence interval, *EIA* Enzyme immunoassay, *FN* False negatives, *FP* False positives, *MBA* Multiplex bead assay, *MeV N* Baculovirus-expressed measles nucleoprotein, *MeV WVAL* Laboratory-produced purified measles whole-virus antigen, *MeV WVA*_*c*_ Commercially produced whole-virus antigen, *MIA* Multiplex immunoassay, *m* Months, *NPV* Negative predictive value, *NE* Not estimable, *NR* Not reported, *O.D* Optical density, *PRNT* Plaque reduction neutralization test, *PPV* Positive predictive value, *R* Correlation coefficient, *TN* True negatives, *TP* True positives, *yrs* years

^a^EIA thresholds reported did not use or did not explicitly report to use manufacturers recommendation

^b^Reported to use methods other than those described in Albrecht et. Al. 1981 or did not describe methodology

^c^Estimates presented were not reported by authors but calculated using data reported

^d^Estimates presented are reported by authors. Estimates could not be re-calculated owing to lack of data

^e^Random EIA subset tested on PRN

^f^Random subset of negative, low positive, medium positive and high positive PRN titers selected for EIA

#### Methodological quality assessment

Based on the QUADAS-2 tool assessment, we concluded there was no bias evident in any of the included studies to justify exclusion (Supplementary Fig. 1). Overall, the intent of the QUADAS-2 tool did not suit the objective of the present review [37] and we used a modified version for methodological quality assessments. However, challenges with applicability of the tool’s domains remained, including inability of reviewers to assess domains when study authors did not report needed information in the text.

### Diagnostic accuracy of EIA assays compared to PRNT

The original intent of this review was to provide a quantitative pooled summary of sensitivity and specificity of EIA results compared to PRNT and evaluate hypothesized risk factors for variability in diagnostic accuracy such as assay type, thresholds used, age of study population, and measles elimination. However, there was an insufficient number of studies per category to identify generalizable patterns.

**Fig. 2:**
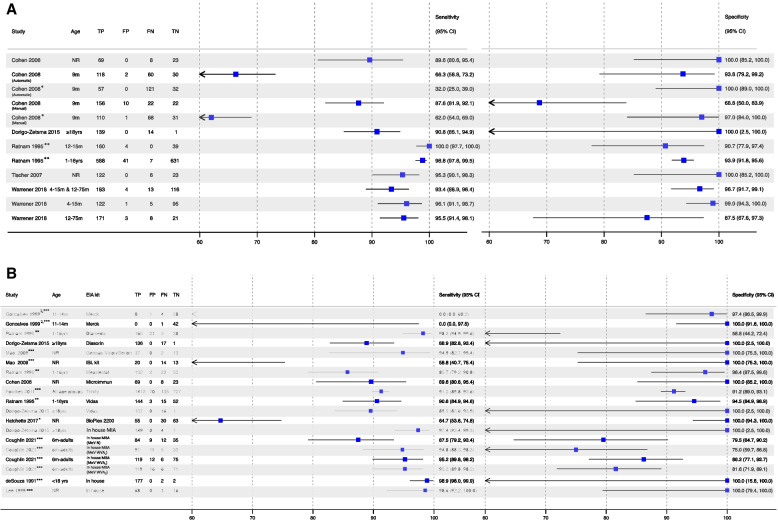
**A** Diagnostic accuracy of Siemens Enzygnost EIA kit compared to PRN reported in high quality studies. % sensitivity and specificity presented. CI, confidence interval. EIA, enzyme immunoassay. FN, false negatives. FP, false positives. NE, not estimable. NR, not reported. TN, true negatives. TP, true positives. *Study classified EIA equivocals as EIA negative. **Study excluded EIA equivocals. All other studies classified EIA equivocals as EIA positive. All studies used the Enzygnost EIA kit with a threshold of < 0.1 O.D (except Warrener where the threshold was not reported). All PRNT tests reported to used a threshold of ≥ 120 mIU/mL except Cohen 2008 which used a batch-specific thresholds and Tischer et al. which reported to use “40 ± 20mIU/mL”. We do not report any comparisons that used EIA thresholds from eg., 8mIU/mL. Cohen 2008 authors reported weighted estimates, unweighted estimates displayed. All papers tested samples by both index and reference tests except Cohen 2008 (both these all samples by PRNT and selected a subset of those samples for EIA testing).” **B** Diagnostic accuracy of non-Siemens Enzygnost EIA kits compared to PRN reported in high quality studies. % sensitivity and specificity presented. CI, confidence interval. EIA, enzyme immunoassay. FN, false negatives. FP, false positives. MBA, Multiplex bead assay. MIA, Multiplex immunoassay. MeV N, recombinant measles virus nucleoprotein. MeV WVAL, Laboratory-produced purified measles whole-virus antigen. MeV WVAc, Commercially produced whole-virus antigen. NE, not estimable. NR, not reported. TN, true negatives. TP, true positives. *Study classified EIA equivocals as EIA negative. **Study excluded EIA equivocals. ***How EIA equivocal were treated was not reported. All other studies classified EIA equivocals as EIA positive. 1)EIA threshold of < 40mIU/mL and PRNT threshold of ≥ 40mIU/mL 2) EIA threshold of < 100mIU/mL and PRNT threshold of ≥ 100mIU/mL. All samples were tested by both index and reference tests but the small number of PRN positive samples by EIA threshold of < 100mIU/mL and PRNT threshold of ≥ 100mIU/mL limited our ability to estimate sensitivity. All PRNT tests reported to used a threshold of ≥ 120 mIU/mL except Goncalves 1999 et al. (at birth age group) used a threshold of 40mIU/mL, Mao 2009 et al. used threshold 1:4 titer, Cohen 2006 et al. used a batch-specific threshold, Lee 1999 et al. used a threshold of > 200mIU/ml, Hatchette 2017 et al. used a threshold of > 192mIU/mL and deSouza 1991 et al. did not report a threshold. EIA equivocals were grouped differently depending on the study. All papers tested samples by index and reference tests except Fowlkes 2011(tested random subset of EIA tested samples by PRNT), Lee 1999 (both these all samples by PRNT and selected a subset of those samples for EIA testing). BioPlex 2200 MMRV IgG is reported as “BioPlex 2200””

Since most high quality studies used Enzygnost, we assessed the sensitivity and specificity of this assay separately and generated pooled diagnostic accuracy estimates. The sensitivity of the Enzygnost EIA ranged from 66.3% to 100.0% with median (IQR) = 92.1 [82.3, 95.7] (Fig. 2A, Table 3A, Supplementary Table 3). Specificity ranged from 68.8% to 100.0% and median (IQR) = 96.9 [93.0, 100.0]. Confidence intervals on specificity were much wider compared to the sensitivity estimates. Seven comparisons reported sensitivities ≥ 90.0%, ten reported specificities ≥ 90.0%, and six reported both sensitivity and specificity of ≥ 90.0% (Fig. 2A and Supplementary Table 3). When high quality studies using the Enzygnost kit were combined in an HSROC curve, the pooled sensitivity and specificity were 91.6% (95%CI: 80.7, 96.6) and 96.0 (95%CI: 90.9, 98.3), respectively (Supplementary Fig. 2).

The sensitivity of all other EIA kits across high quality studies ranged from 0% to 98.9% with median (IQR) = 90.6 [86.6, 95.2] (Fig. 2B, Table 4, Supplementary Table 3). The specificity of all other EIA kits across high quality studies ranged from 58.8% to 100.0% with median (IQR) = 100.0 [88.7, 100.0]. When studies with fewer than five PRNT seropositive individuals[48] were excluded (*n* = 1), the sensitivity of all other EIA kits ranged from 58.8% to 98.9% (Fig. 2B). Ten comparisons reported sensitivities ≥ 90.0%, fourteen reported specificities ≥ 90.0%, and six reported both sensitivity and specificity ≥ 90.0% (Fig. 2B and Supplementary Table 3). There were no observed differences in median sensitivity or specificity by study quality (Supplementary Table 3).

In addition to Enzygnost, the VIDAS, DiaSorin LIAISON®, and commercial MBA EIA kits were used in at least three comparisons, allowing general assessments of within-kit performance across studies without stratifying by quality classification (Supplementary Fig. 4 and Supplementary Table 3). The three sensitivity estimates for the Diasorin assay were overall slightly lower than Enzygnost, ranging from 87.2% to 90.2%, with variable specificity (75.0% to 100%). Sensitivity estimates from three of the four studies using the VIDAS assay were comparable to Enzygnost (87.2% to 90.6%) with less variability in specificity (86.4% to 100%). Although in-house EIAs are all different, each study except one reported high sensitivity (86.8% to 100%) and specificity (80% for one study, 100% for all others). Calculated sensitivity was more variable for MBAs compared to Enzygnost, VIDAS, and DiaSorin. Insufficient information on MBA assays was available to assess reasons for variability.

#### Sensitivity analysis when reclassifying equivocal results

For studies that provided sufficient information regarding how equivocal EIA results were classified, we regrouped equivocal results to assess how this classification affected the sensitivity and specificity. Five high quality studies and one medium quality study included sufficient information for reclassification (Supplementary Fig. 5), in which 2% to 30% of samples tested had equivocal results. Equivocal EIA results were analyzed in the primary analyses as they were reported in the original publication: three studies grouped equivocal results as positive, one grouped equivocal results as negative, one reported results treating equivocal results as both positive and negative, and one excluded equivocal results from the analysis. As expected, sensitivity metrics increased when EIA equivocal results were grouped with positives. Compared to when equivocal results were excluded or grouped with negatives, sensitivity increased by up to 35.3 percentage points when grouped with positives. Conversely, specificity metrics increased when EIA equivocal results were grouped with negative. Compared to when equivocal results were excluded or grouped with positives, specificity was increased up to 36.5 percentage points when grouped with negatives.

## Discussion

Serosurveillance has the potential to be a powerful tool for informing vaccination program design and monitoring. Historically, cross-sectional seroprevalence studies have contributed to epidemiological understanding of poliomyelitis, rubella, and hepatitis A and B virus infections [69]. More recently, serosurveillance has been used globally as a method for monitoring population immunity against HIV [70, 71] and SARS-CoV-2 [72–75], and biomarkers are increasingly included in country-level household surveys such as the Demographic and Health Surveys (DHS) and the National Health and Nutrition Examination survey (NHANES) [76–78]. As countries continue to work towards measles elimination, if antibody prevalence can be accurately measured and subsequently correlates with immunity, then serosurveillance can contribute to monitoring progress, identifying gaps in population immunity and susceptible segments of the population, understanding reasons for apparent increases in incidence and resurgence of disease, and evaluating vaccine impact [12, 69, 79, 80]. This is particularly important after declines in routine immunization rates globally during the COVID pandemic. EIA assays are less resource-intensive and require less technical expertise than the current gold standard PRNTs and are widely used in laboratories around the world including low-income countries, and as such can be deployed on the larger scale. Given the relative ease of these assays, establishing their diagnostic accuracy is important for broader use in research and surveillance.

This analysis summarized and, to the extent possible, compared the diagnostic accuracy of measles IgG EIA assays to gold standard PRNT. Overall, the sensitivity of measles IgG antibody EIAs were moderate to excellent, but highly variable. Specificity tended to be lower and estimates were often imprecise due to the small number of seronegative individuals. With the exception of studies evaluating the Enzygnost EIA, there were an insufficient number of comparable studies to generalize on the diagnostic accuracy of other EIAs compared to PRNT. Studies were too diverse in terms of age groups, population characteristics (e.g., vaccination status, measles endemicity), EIA kits used, EIA and PRNT threshold(s) used, treatment of EIA equivocal results, and inclusion/exclusion of samples for testing to allow a meta-analysis or a more systematic analysis of factors associated with diagnostic accuracy. Measles vaccination status of the mother was not available for studies with young children. Furthermore, the lack of standardization of methods and reporting of results, even among studies that explicitly sought to assess diagnostic accuracy, limited our ability to make meaningful inferences regarding the performance of EIA kits.

Optimal diagnostic accuracy characteristics depend on the objective of the activity, risk of misclassification, and consequences and cost of the subsequent intervention. For example, diagnostic testing at the individual level (e.g., HIV testing) or case detection early in outbreaks settings aim to minimize false negatives by maximizing sensitivity. For seroprevalence studies, misclassification in either direction could result in important public health consequences and should be considered. The general priority for measles serosurveillance is to identify susceptible populations to assess progress toward elimination or trigger supplemental activities to fill immunity gaps. Assays that are inadequately specific could result in overestimates of population immunity, leaving susceptible individuals at risk and may result in large, unexpected measles outbreaks that could have been prevented. On the other hand, assays that are inadequately sensitive would underestimate population immunity, which could lead to unnecessary and costly supplementary immunization activities [30]. Hence, an important limitation to consider is that EIAs are designed for determining individual immunity. As such, they err on the side of high specificity (i.e., minimizing false positives, the lesser risk to the individual being to classify someone who is immune as susceptible, rather than classifying someone who is susceptible as immune) and may not be fit for purposes related to population-level serosurveillance. It would therefore be useful to better characterize acceptable diagnostic accuracy thresholds for EIAs when adapted for use in such contexts.

Our results revealed substantial variability in test performance of measles EIAs and may help to contextualize the results of recent large scale measles serosurveys in Laos [81], Bhutan [30], Zambia [82], Madagascar [83], Canada [41], and the Democratic Republic of Congo (DRC) [76, 84]. Results from some of these studies were unexpected and speak to the importance of validating the diagnostic accuracy of measles IgG antibody EIA kits. The serosurveys from Canada (2019) and Bhutan (2017) conducted validation testing via PRNT on a subset of EIA seronegative and equivocal results, which demonstrated a non-negligible proportion were positive by PRNT (33.3% and 10%, respectively). A serosurvey in Laos reported relatively low seroprevalence among children ages one to two years (48.6%), which was substantially lower than expected based on estimated vaccination coverage of 69% to 72% and was also lower than in persons aged 5–21 years (86.8%). Validation of a random sample of results, or a subset of seronegative results using PRNT would have helped to understand the discrepancies.

Evaluation and interpretation of measles EIA results can be complex, particularly in measles elimination settings where antibody levels are not boosted by exposure to wild-type measles virus. False negative results and over-estimation of population susceptibility are risks with EIAs in elimination settings. Average antibody concentrations are likely to have waned, but individuals could still mount an anamnestic antibody response if exposed to measles, which has important consequences for the working definition of “susceptible”. Twelve studies included in this analysis, most of which were population-based seroprevalences studies, attempted to better characterize EIA accuracy near the threshold by testing all or a sample of EIA negative, equivocal, or low-positive samples by PRNT.

An in-depth head-to-head analysis of commercially available measles IgG assays would help to build confidence in large-scale measles serosurveys and surveillance programs. A recent study conducted in the United States performed head-to-head comparisons of five commercial EIAs with PRN and found discrepant results for samples in the low-positive ranges of even the most sensitive EIAs [53]. This study was included in this review but was classified as medium-quality because information needed to generate two-by-two tables were not included. False negative EIA test results occurred in approximately 11% of sera, which generally had low levels of neutralizing antibody. The study demonstrated that lowering the PRN threshold (i.e., rather than the EIA threshold) from 120 to 40 mIU/mL increased specificity of EIA assays at the expense of sensitivity. Although there is debate on the 120 PRN correlate of protection, lowering the threshold to 40 mIU/mL is unlikely to be clinically meaningful [85].

In addition, systematic analyses of diagnostic accuracy among vaccinated populations, of varying ages, in elimination settings, where average antibody levels are generally low, would help to fill evidence gaps identified in this review. Alternatives to traditional EIAs, such as MBAs, have demonstrated excellent diagnostic accuracy and analytic sensitivity for other disease-specific antibodies and are promising for measles serosurveillance [86, 87]. However, limitations in access to multiplex machines, availability of commercially available regents with measles antigens, and cost limit their used in low- and lower-middle income settings. Promising microneutralization assays may also overcome challenges of evaluating functional responses in surveillance settings [88, 89].

## Strengths and limitations

This review included studies conducted between 1984 and 2020, over which time diagnostic and analytic methods have changed, limiting conclusions we can draw about EIAs in contemporary use. For example, Enzgynost EIA was the most common assay used in included studies, but was recently discontinued [90]. The EUROIMMUN Anti-Measles Virus IgG ELISA is used frequently in seroprevalence studies at present [91, 92], but was not assessed in any studies returned from the searches and therefore not included in this review.

This review contributes to the existing literature on EIA and PRNT diagnostic accuracy for the identification of measles IgG and is the first to systematically review their comparative test performance. It identified critical gaps regarding systematic reporting and use of standardized methodologies. The literature search was not limited by language but translated full-texts were not available for three publications and may have limited the analysis.

## Conclusions

To expand the utility of measles serological surveillance, robust, feasible, high-throughput, and accurate assays are needed to identify susceptible and protection populations. Evidence on the diagnostic accuracy of currently available measles IgG EIAs is variable, insufficient, and may not be fit for purpose for serosurveillance goals. Additional studies evaluating the diagnostic accuracy of measles EIAs, including MBAs, should be conducted among diverse populations and settings (e.g., vaccination status, elimination/endemic status, age groups). Analyses of serosurveys would be strengthened if PRNT validation were conducted on a random subsample or on samples near the EIA threshold.

## Supplementary Information

**Additional file 1:** **Supplemental Table 1.** PRISMA statement for a systematic literature search checklist. **Supplemental Table 2A.** Studies evaluating EIA compared to PRNT. **Supplemental Table 2B.** Studies evaluating EIA compared to PRNT. **Supplementary Table 3.** Mediandiagnostic accuracy of EIA compared to PRNT by assay type and study quality. **Supplementary Table 4.** Diagnostic accuracy measures reported in medium quality studies. **Supplementary Figure 1.** Summary of Quality Assessment of Diagnostic Accuracy Studiesresults. **Supplementary Figure 2.** HSROC curves for measles EIA acompared to PRNT for high quality studies evaluating Siemens Enzygnost EIA kits. **Supplementary Figure 3.** Diagnostic accuracy of EIA compared to PRN reported in medium quality studies. **Supplementary Figure 4.** Diagnostic accuracy of EIA assays compared to PRN by assay type. **Supplementary Figure 5.** Diagnostic accuracy of EIA compared to PRNT when EIA equivocals are re-classified, compared to results reported in high quality studies.

## Data Availability

All data is available publicly via PubMed and Embase search engines; search strategies have been preserved on searchRxiv (https://searchrxiv.org/). Abstraction tools, generated datasets, and programs and code used for statistical analyses can be made available upon reasonable request to the corresponding author.

## Abbreviations

CI: 95% Confidence interval
EIA: Enzyme immunosorbent assay
HSROC: Hierarchical summary receiver operating characteristic
IgG: Immunoglobulin G
MCV1: Measles-containing vaccine, dose 1
MCV2: Measles-containing vaccine, dose 2
MBA: Multiplexed bead assay
NPV: Negative predictive value
NT: Neutralizing test
PRNT: Plaque reduction neutralization test
PPV: Positive predictive value
QUADAS: Quality Assessment of Diagnostic Accuracy Studies
SDTM: Cochrane Screening and Diagnostic Tests Methods Group
STARD: Standards for Reporting of Diagnostic Accuracy Studies

## References

[1] Moss WJ (2017). Measles. Lancet.

[2] Dixon MG, Ferrari M, Antoni S, Li X, Portnoy A, Lambert B (2021). Progress Toward Regional Measles Elimination - Worldwide, 2000–2020. MMWR Morb Mortal Wkly Rep.

[3] World Health Organization. Measles vaccination coverage 2021 [Available from: https://immunizationdata.who.int/pages/coverage/mcv.html?CODE=Global&ANTIGEN=MCV2&YEAR=.

[4] World Health Organization. Introduction of Measles-containing vaccine 2nd dose 2021 [Available from: https://immunizationdata.who.int/pages/vaccine-intro-by-antigen/mcv2.html?ISO_3_CODE=&YEAR=.

[5] World Health Organization. Measles: fighting a global resurgence 2019 [Available from: https://www.who.int/news-room/feature-stories/detail/measles-fighting-a-global-resurgence.

[6] Measles cases spike globally due to gaps in vaccination coverage [press release]. https://www.who.int/news/item/29-11-2018-measles-cases-spike-globally-due-to-gaps-in-vaccination-coverage. Accessed 23 May 2023.

[7] World Health Organization. Measles reported cases and incidence 2021 [Available from: https://immunizationdata.who.int/pages/incidence/measles.html?CODE=Global&YEAR=.

[8] Patel MK, Goodson JL, Alexander JP, Kretsinger K, Sodha SV, Steulet C (2020). Progress Toward Regional Measles Elimination - Worldwide, 2000–2019. MMWR Morb Mortal Wkly Rep.

[9] World Health Organization. Nearly 40 million children are dangerously susceptible to growing measles threat 2022 Nov 23 [Available from: https://www.who.int/news/item/23-11-2022-nearly-40-million-children-are-dangerously-susceptible-to-growing-measles-threat.

[10] Lessler J, Metcalf CJ, Cutts FT, Grenfell BT (2016). Impact on Epidemic Measles of Vaccination Campaigns Triggered by Disease Outbreaks or Serosurveys: A Modeling Study. PLoS Med.

[11] Andrews N, Tischer A, Siedler A, Pebody RG, Barbara C, Cotter S (2008). Towards elimination: measles susceptibility in Australia and 17 European countries. Bull World Health Organ.

[12] Cutts FT, Hanson M (2016). Seroepidemiology: an underused tool for designing and monitoring vaccination programmes in low- and middle-income countries. Trop Med Int Health.

[13] Klasse PJ (2014). Neutralization of Virus Infectivity by Antibodies: Old Problems in New Perspectives. Adv Biol.

[14] Albrecht P, Herrmann K, Burns GR (1981). Role of virus strain in conventional and enhanced measles plaque neutralization test. J Virol Methods.

[15] Griffin DE, Knipe DM, Howley PM (2001). Measles virus. Fields virology.

[16] Science M, Savage R, Severini A, McLachlan E, Hughes SL, Arnold C (2019). Measles Antibody Levels in Young Infants. Pediatrics.

[17] Chen RT, Markowitz LE, Albrecht P, Stewart JA, Mofenson LM, Preblud SR (1990). Measles antibody: reevaluation of protective titers. J Infect Dis.

[18] Plotkin SA (2008). Vaccines: correlates of vaccine-induced immunity. Clin Infect Dis.

[19] Coughlin MM, Matson Z, Sowers SB, Priest JW, Smits GP, van der Klis FRM (2021). Development of a Measles and Rubella Multiplex Bead Serological Assay for Assessing Population Immunity. J Clin Microbiol.

[20] Cohen BJ, Audet S, Andrews N, Beeler J, test WHOwgomprn (2007). Plaque reduction neutralization test for measles antibodies: Description of a standardised laboratory method for use in immunogenicity studies of aerosol vaccination. Vaccine.

[21] Cohen BJ, Doblas D, Andrews N (2008). Comparison of plaque reduction neutralisation test (PRNT) and measles virus-specific IgG ELISA for assessing immunogenicity of measles vaccination. Vaccine.

[22] Coughlin MM, Beck AS, Bankamp B, Rota PA (2017). Perspective on Global Measles Epidemiology and Control and the Role of Novel Vaccination Strategies. Viruses.

[23] Immunological Basis for Immunization Series (2009). Measles - Update 2009.

[24] Cho HK, Lee H, Kim HW, Kim SS, Kang HJ, Kim IT (2016). Seroprevalences of Specific IgG Antibodies to Measles, Mumps, and Rubella in Korean Infants. J Korean Med Sci.

[25] Cohen BJ, Parry RP, Doblas D, Samuel D, Warrener L, Andrews N (2006). Measles immunity testing: comparison of two measles IgG ELISAs with plaque reduction neutralisation assay. J Virol Methods.

[26] Hatchette TF, Scholz H, Bolotin S, Crowcroft NS, Jackson C, McLachlan E (2017). Calibration and Evaluation of Quantitative Antibody Titers for Measles Virus by Using the BioPlex 2200. Clin Vaccine Immunol.

[27] Tischer A, Gassner M, Richard JL, Suter-Riniker F, Mankertz A, Heininger U (2007). Vaccinated students with negative enzyme immunoassay results show positive measles virus-specific antibody levels by immunofluorescence and plaque neutralisation tests. J Clin Virol.

[28] World Health Organization. Africa Regional Guidelines for Measles and Rubella Surveillance. WHO Regional Office for Africa: WHO Regional Office for Africa; 2015.

[29] World Health Organization. Guidance on Conducting Serosurveys in Support of Measles and Rubella Elimination in the WHO European Region. WHO Regional Office for Europe: WHO Regional Office for Europe; 2013. https://apps.who.int/iris/handle/10665/350485.

[30] Wangchuk S, Nogareda F, Tshering N, Khandu L, Pelden S, Wannemuehler K (2019). Measles and rubella immunity in the population of Bhutan, 2017. Vaccine.

[31] Liberati A, Altman DG, Tetzlaff J, Mulrow C, Gotzsche PC, Ioannidis JP (2009). The PRISMA statement for reporting systematic reviews and meta-analyses of studies that evaluate healthcare interventions: explanation and elaboration. BMJ.

[32] Page MJ, McKenzie JE, Bossuyt PM, Boutron I, Hoffmann TC, Mulrow CD (2021). The PRISMA 2020 statement: an updated guideline for reporting systematic reviews. BMJ.

[33] The Cochrane Library. Cochrane screening and diagnostic tests methods group (SDTM) 2020 [Available from: https://methods.cochrane.org/sdt/welcome.

[34] Covidence systematic review software, Veritas Health Innovation, Melbourne, Australia. [Available from: www.covidence.org.

[35] Cohen JF, Korevaar DA, Altman DG, Bruns DE, Gatsonis CA, Hooft L (2016). STARD 2015 guidelines for reporting diagnostic accuracy studies: explanation and elaboration. BMJ Open.

[36] Smit PW, Elliott I, Peeling RW, Mabey D, Newton PN (2014). An overview of the clinical use of filter paper in the diagnosis of tropical diseases. Am J Trop Med Hyg.

[37] Whiting PF, Rutjes AW, Westwood ME, Mallett S, Deeks JJ, Reitsma JB (2011). QUADAS-2: a revised tool for the quality assessment of diagnostic accuracy studies. Ann Intern Med.

[38] StataCorp (2019). Stata Statistical Software: Release 16.

[39] Al-Mazrou YY, Khalil MK, Tischer A, Al-Jeffri MH, Al-Ghamdi YS, Bakhsh MM (2005). Serosurvey of measles, mumps and rubella antibodies in Saudi children. Saudi Med J.

[40] Al-Mazrou YY, Khalil MK, Tumsah S, Al-Ghamdi YS, Al-Jeffri MH, Tischer AB (2002). Sero-response to measles-mumps-rubella vaccine campaign in Saudi Arabia. Saudi Med J.

[41] Bolotin S, Severini A, Hatchette T, McLachlan E, Savage R, Hughes SL (2019). Assessment of population immunity to measles in Ontario, Canada: a Canadian Immunization Research Network (CIRN) study. Hum Vaccin Immunother.

[42] Castro-Silva R, Camacho LA, Amorim L, Medeiros AD, Ferreira DA, Oliveira SA (2003). Serological surveillance of measles in blood donors in Rio de Janeiro. Brazil Rev Panam Salud Publica.

[43] Cremer NE, Cossen CK, Shell G, Diggs J, Gallo D, Schmidt NJ (1985). Enzyme immunoassay versus plaque neutralization and other methods for determination of immune status to measles and varicella-zoster viruses and versus complement fixation for serodiagnosis of infections with those viruses. J Clin Microbiol.

[44] de Souza VA, Pannuti CS, Sumita LM, Albrecht P (1991). Enzyme-linked immunosorbent assay (ELISA) for measles antibody. A comparison with haemagglutination inhibition, immunofluorescence and plaque neutralization tests. Rev Inst Med Trop Sao Paulo.

[45] Dorigo-Zetsma JW, Leverstein-van Hall MA, Vreeswijk J, de Vries JJ, Vossen AC, Ten Hulscher HI (2015). Immune status of health care workers to measles virus: evaluation of protective titers in four measles IgG EIAs. J Clin Virol.

[46] Erdman DD, Anderson LJ, Adams DR, Stewart JA, Markowitz LE, Bellini WJ (1991). Evaluation of monoclonal antibody-based capture enzyme immunoassays for detection of specific antibodies to measles virus. J Clin Microbiol.

[47] Fowlkes A, Witte D, Beeler J, Audet S, Garcia P, Curns A (2011). Persistence of vaccine-induced measles antibody beyond age 12 months: a comparison of response to one and two doses of Edmonston-Zagreb measles vaccine among HIV-infected and uninfected children in Malawi. J Infect Dis.

[48] Gonçalves G, Cutts F, Forsey T, Andrade HR (1999). Comparison of a commercial enzyme immunoassay with plaque reduction neutralization for maternal and infant measles antibody measurement. Rev Inst Med Trop Sao Paulo.

[49] Hesketh L, Charlett A, Farrington P, Miller E, Forsey T, Morgan-Capner P (1997). An evaluation of nine commercial EIA kits for the detection of measles specific IgG. J Virol Methods.

[50] Job JS, Halsey NA, Boulos R, Holt E, Farrell D, Albrecht P (1991). Successful immunization of infants at 6 months of age with high dose Edmonston-Zagreb measles vaccine. Cite Soleil/JHU Project Team. Pediatr Infect Dis J.

[51] Kang HJ, Han YW, Kim SJ, Kim YJ, Kim AR, Kim JA (2017). An increasing, potentially measles-susceptible population over time after vaccination in Korea. Vaccine.

[52] Kidokoro M, Aoki A, Horiuchi K, Shida H (2002). Large-scale preparation of biologically active measles virus haemagglutinin expressed by attenuated vaccinia virus vectors. Microbes Infect.

[53] Latner DR, Sowers SB, Anthony K, Colley H, Badeau C, Coates J (2020). Qualitative Variation among Commercial Immunoassays for Detection of Measles-Specific IgG. J Clin Microbiol.

[54] Lee MS, Cohen B, Hand J, Nokes DJ (1999). A simplified and standardized neutralization enzyme immunoassay for the quantification of measles neutralizing antibody. J Virol Methods.

[55] Mancuso JD, Krauss MR, Audet S, Beeler JA (2008). ELISA underestimates measles antibody seroprevalence in US military recruits. Vaccine.

[56] Mao NY, Zhu Z, Jiang XH (2009). Comparison and evaluation of enzyme-linked immunization assay kits with plague reduction neutralization test for detection of measles IgG antibody. Zhongguo Yi Miao He Mian Yi.

[57] Matson DO, Byington C, Canfield M, Albrecht P, Feigin RD (1993). Investigation of a measles outbreak in a fully vaccinated school population including serum studies before and after revaccination. Pediatr Infect Dis J.

[58] Morris LE, Posada R, Hickman CJ, Latner DR, Singh TA, Rautenberg A (2015). Susceptibility to Measles Among Perinatally HIV-Infected Adolescents and Young Adults. J Pediatric Infect Dis Soc.

[59] Ng Y, Chua LAV, Cui L, Ang LW, Tee NWS, Lin RTP (2020). Seroprevalence of vaccine-preventable diseases among children and adolescents in Singapore: Results from the National Paediatric Seroprevalence Survey 2018. Int J Infect Dis.

[60] Oliveira SA, Siqueira MM, Mann GF, Costa AJ, Almeida MT, Stavola MS (1996). Measles antibody prevalence after mass immunization campaign in Niterói, state of Rio de Janeiro, Brazil. Rev Inst Med Trop Sao Paulo.

[61] Pabst HF, Spady DW, Carson MM, Krezolek MP, Barreto L, Wittes RC (1999). Cell-mediated and antibody immune responses to AIK-C and Connaught monovalent measles vaccine given to 6 month old infants. Vaccine.

[62] Pourabbas B, Ziyaeyan M, Alborzi A, Mardaneh J (2008). Efficacy of measles and rubella vaccination one year after the nationwide campaign in Shiraz. Iran Int J Infect Dis.

[63] Ratnam S, Gadag V, West R, Burris J, Oates E, Stead F (1995). Comparison of commercial enzyme immunoassay kits with plaque reduction neutralization test for detection of measles virus antibody. J Clin Microbiol.

[64] Siennicka J, Częścik A, Trzcińska A (2014). The significance for epidemiological studies anti-measles antibody detection examined by enzyme immunoassay (EIA) and plaque reduction neutralization test (PRNT). Przegl Epidemiol.

[65] Tapia MD, Sow SO, Medina-Moreno S, Lim Y, Pasetti MF, Kotloff K (2005). A serosurvey to identify the window of vulnerability to wild-type measles among infants in rural Mali. Am J Trop Med Hyg.

[66] Tischer A, Andrews N, Kafatos G, Nardone A, Berbers G, Davidkin I (2007). Standardization of measles, mumps and rubella assays to enable comparisons of seroprevalence data across 21 European countries and Australia. Epidemiol Infect.

[67] Warrener L, Bwogi J, Andrews N, Samuel D, Kabaliisa T, Bukenya H (2018). Serum anti-tetanus and measles antibody titres in Ugandan children aged 4 months to 6 years: implications for vaccine programme. Epidemiol Infect.

[68] Weigle KA, Murphy MD, Brunell PA (1984). Enzyme-linked immunosorbent assay for evaluation of immunity to measles virus. J Clin Microbiol.

[69] Osborne K, Gay N, Hesketh L, Morgan-Capner P, Miller E (2000). Ten years of serological surveillance in England and Wales: methods, results, implications and action. Int J Epidemiol.

[70] Endres-Dighe S, Farris T, Courtney L (2018). Lessons learned from twelve years of HIV Seroprevalence and Behavioral Epidemiology Risk Survey (SABERS) development and implementation among foreign militaries. PLoS ONE.

[71] National Bureau of Statistics - Nigeria. National HIV Sero-Prevalence Sentinel Survey 2008, Eight rond. Federal Ministry of Health - Federal Government of Nigeria: Federal Ministry of Health - Federal Government of Nigeria; 2020 Jul 11.

[72] Aziz NA, Corman VM, Echterhoff AKC, Muller MA, Richter A, Schmandke A (2021). Seroprevalence and correlates of SARS-CoV-2 neutralizing antibodies from a population-based study in Bonn, Germany. Nat Commun.

[73] Kindgen-Milles D, Brandenburger T, Braun JFW, Cleff C, Moussazadeh K, Mrosewski I (2021). Prevalence of SARS-COV-2 positivity in 516 German intensive care and emergency physicians studied by seroprevalence of antibodies National Covid Survey Germany (NAT-COV-SURV). PLoS ONE.

[74] Pasqualotto AC, Pereira PC, Lana DFD, Schwarzbold AV, Ribeiro MS, Riche CVW (2021). COVID-19 seroprevalence in military police force, Southern Brazil. PLoS ONE.

[75] Mulenga LB, Hines JZ, Fwoloshi S, Chirwa L, Siwingwa M, Yingst S (2021). Prevalence of SARS-CoV-2 in six districts in Zambia in July, 2020: a cross-sectional cluster sample survey. Lancet Glob Health.

[76] Ashbaugh HR, Cherry JD, Hoff NA, Doshi RH, Alfonso VH, Gadoth A (2020). Measles antibody levels among vaccinated and unvaccinated children 6–59months of age in the Democratic Republic of the Congo, 2013–2014. Vaccine.

[77] McQuillan GM, Kruszon-Moran D, Granade T, Feldman JW (2010). Seroprevalence of HIV in the US Household Population Aged 18–49 Years: The National Health and Nutrition Examination Surveys, 1999–2006. J Acquir Immune Defic Syndr.

[78] Mishra V, Medley A, Hong R, Gu Y, Robey B. Levels and spread of HIV seroprevalence and associated factors: evidence from national household surveys. New York: DHS Comparative Reports 22: United States Agency for International Development (USAID); 2009.

[79] Wilson SE, Deeks SL, Hatchette TF, Crowcroft NS (2012). The role of seroepidemiology in the comprehensive surveillance of vaccine-preventable diseases. CMAJ.

[80] Winter AK, Martinez ME, Cutts FT, Moss WJ, Ferrari MJ, McKee A (2018). Benefits and Challenges in Using Seroprevalence Data to Inform Models for Measles and Rubella Elimination. J Infect Dis.

[81] Hachiya M, Miyano S, Mori Y, Vynnycky E, Keungsaneth P, Vongphrachanh P (2018). Evaluation of nationwide supplementary immunization in Lao People's Democratic Republic: Population-based seroprevalence survey of anti-measles and anti-rubella IgG in children and adults, mathematical modelling and a stability testing of the vaccine. PLoS One.

[82] Hayford K, Mutembo S, Carcelen A, Matakala HK, Munachoonga P, Winter A (2019). Measles and rubella serosurvey identifies rubella immunity gap in young adults of childbearing age in Zambia: The added value of nesting a serological survey within a post-campaign coverage evaluation survey. Vaccine.

[83] Winter AK, Wesolowski AP, Mensah KJ, Ramamonjiharisoa MB, Randriamanantena AH, Razafindratsimandresy R (2018). Revealing Measles Outbreak Risk With a Nested Immunoglobulin G Serosurvey in Madagascar. Am J Epidemiol.

[84] Keating P, Carrion Martin AI, Blake A, Lechevalier P, Uzzeni F, Gignoux E (2019). Measles seroprevalence after reactive vaccination campaigns during the 2015 measles outbreak in four health zones of the former Katanga Province, Democratic Republic of Congo. BMC Public Health.

[85] Bolotin S, Hughes SL, Gul N, Khan S, Rota PA, Severini A (2020). What Is the Evidence to Support a Correlate of Protection for Measles? A Systematic Review. J Infect Dis.

[86] Binnicker MJ, Jespersen DJ, Harring JA, Rollins LO, Beito EM (2008). Evaluation of a multiplex flow immunoassay for detection of epstein-barr virus-specific antibodies. Clin Vaccine Immunol.

[87] Fonseca BP, Marques CF, Nascimento LD, Mello MB, Silva LB, Rubim NM (2011). Development of a multiplex bead-based assay for detection of hepatitis C virus. Clin Vaccine Immunol.

[88] Alvarado-Facundo E, Audet S, Moss WJ, Beeler JA (2019). Development of a high-throughput assay to measure measles neutralizing antibodies. PLoS ONE.

[89] Knipes AK, Summers A, Sklavounos AA, Lamanna J, de Campos RPS, Narahari T (2022). Use of a rapid digital microfluidics-powered immunoassay for assessing measles and rubella infection and immunity in outbreak settings in the Democratic Republic of the Congo. PLoS ONE.

[90] DiaSorin. 25 July 2017. Press release: DiaSorin to acquire ELISA immunodiagnostic business portfolio and associated assets from Siemens Healthineers. https://diasoringroup.com/sites/diasorincorp/files/allegati_pressrel/pr_diasorin_-_acquisition_of_siemens_elisa_eng_1.pdf. Accessed 2021 Nov 22 [press release].

[91] Doornekamp L, Comvalius AD, GeurtsvanKessel CH, Slobbe L, Scherbeijn SMJ, van Genderen PJJ (2021). Measles seroprevalence among Dutch travelling families. Travel Med Infect Dis.

[92] Meng QH, Liu Y, Yu JQ, Li LJ, Shi W, Shen YJ (2018). Seroprevalence of Maternal and Cord Antibodies Specific for Diphtheria, Tetanus, Pertussis, Measles, Mumps and Rubella in Shunyi, Beijing. Sci Rep.

