# Supplementary material for: Comparison of measles IgG enzyme immunoassays (EIA) versus plaque reduction neutralization test (PRNT) for measuring measles serostatus: a systematic review of head-to-head analyses of measles IgG EIA and PRNT
Source: BMC Infect Dis. 2023 May 31;23:367. doi: 10.1186/s12879-023-08199-8 (PMC10231861; doi:10.1186/s12879-023-08199-8)
Supplement: Supplementary file 1 — Additional file 1: Supplemental Table 1. PRISMA statement for a systematic literature search checklist. Supplemental Table 2A. Studies evaluating EIA compared to PRNT. Supplemental Table 2B. Studies evaluating EIA compared to PRNT. Supplementary Table 3. Mediandiagnostic accuracy of EIA compared to PRNT by assay type and study quality. Supplementary Table 4. Diagnostic accuracy measures reported in medium quality studies. Supplementary Figure 1. Summary of Quality Assessment of Diagnostic Accuracy Studiesresults. Supplementary Figure 2. HSROC curves for measles EIA acompared to PRNT for high quality studies evaluating Siemens Enzygnost EIA kits. Supplementary Figure 3. Diagnostic accuracy of EIA compared to PRN reported in medium quality studies. Supplementary Figure 4. Diagnostic accuracy of EIA assays compared to PRN by assay type. Supplementary Figure 5. Diagnostic accuracy of EIA compared to PRNT when EIA equivocals are re-classified, compared to results reported in high quality studies. [file 12879_2023_8199_MOESM1_ESM.docx]

| **Supplemental Table 1. PRISMA statement for a systematic literature search checklist.** | | |
| --- | --- | --- |
| **Section and topic** | **Item no** | **Checklist item** |
| **ADMINISTRATIVE INFORMATION** | | |
| Title: |  |  |
| Identification | 1a | Protocol for *Comparison of measles IgG enzyme immunoassays (EIA) versus plaque reduction neutralization assay (PRNT) for measuring measles serostatus: A systematic review of head-to-head analyses of measles IgG EIA and PRNT.* |
| Update | 1b | Not applicable. |
| Registration | 2 | Planned submission to PROSPERO February 2020. |
| Authors: |  |  |
| Contact | 3a | Shelly Bolotin, PhD, MSc, MScPH  Communicable Diseases and Emergency Preparedness and Response, Public Health Ontario, Dalla Lana School of Public Health, University of Toronto, 661 University Ave, Toronto, ON M5G 1M1  Email: [Shelly.bolotin@utoronto.ca](mailto:Shelly.bolotin@utoronto.ca)  Natasha Crowcroft, MD(Cantab), MSc, MRCP, FFPH  Immunisation, Vaccines, and Biologicals, World Health Organization, Geneva, Switzerland, 1211  Email: [crowcroftn@who.int](mailto:crowcroftn@who.int)  Felicity Cutts, MD  London School of Hygiene and Tropical Medicine, University of London, Keppel St, Bloomsbury, London, WC1E 7HT, UK  Email: [Felicity.cutts@lshtm.ac.uk](mailto:Felicity.cutts@lshtm.ac.uk)  Alvira Z. Hasan, MSPH  International Vaccine Access Center, Bloomberg School of Public Health, Johns Hopkins University, 415 N. Washington St, 5th Floor, Baltimore, MD 21231  Email: [ahasan7@jhmi.edu](mailto:ahasan7@jhmi.edu)  Kyla Hayford, PhD  Department of International Health, Bloomberg School of Public Health, Johns Hopkins University, 415 N. Washington St, 5th Floor, Baltimore, MD 21231  Phone: 202-657-5318  Email: [kylahayford@jhu.edu](mailto:kylahayford@jhu.edu)  Eugene Joh, MS, MPH  Communicable Diseases and Emergency Preparedness and Response, Public Health Ontario, 661 University Ave, Toronto, ON M5G 1M1  Email: [Eugene.Joh@oahpp.ca](mailto:Eugene.Joh@oahpp.ca)  Stacie Loisate, MSPH  Department of Epidemiology, Bloomberg School of Public Health, Johns Hopkins University, 615 N. Wolfe St, Baltimore, MD 21205  Email: [sloisat1@jhmi.edu](mailto:sloisat1@jhmi.edu)  Chelsea S. Lutz, MPH, CPH  Department of International Health, Bloomberg School of Public Health, Johns Hopkins University, 615 N. Wolfe St, Baltimore, MD 21205  Email: [Chelsea.lutz@jhu.edu](mailto:Chelsea.lutz@jhu.edu)  William J. Moss, MD  International Vaccine Access Center, Departments of Epidemiology, International Health, and Molecular Microbiology and Immunology, Bloomberg School of Public Health, Johns Hopkins University, 615 N. Wolfe St, Baltimore, MD 21205  Email: [Wmoss1@jhu.edu](mailto:Wmoss1@jhu.edu)  Selma Osman, MPH  Applied Immunization Research and Evaluation Program, Public Health Ontario, 480 University Ave, Suite 300, Toronto, ON M5G 1V2  Email: [selma.osman@oahpp.ca](mailto:selma.osman@oahpp.ca) |
| Contributions | 3b | SB, NC, FC, KH, and WM developed original research question. CSL and KH developed original concept note. CSL prepared protocol; KH, SB, and FC commented on all stages of protocol development. CSL and EJ will screen titles/abstracts and full text articles and complete data abstraction. SB, NC, FC, KH, EJ, CSL, and WM will contribute to interpretation of findings. Guarantor of the review: KH. |
| Amendments | 4 | Amendments to the protocol, if needed, will be documented separately and made available in an appendix. |
| Support: |  |  |
| Sources | 5a | CSL is a student research assistant and receives support through Federal Work Study. This project is also supported through a grant by the Bill & Melinda Gates Foundation, which in part supports KH, CSL, and WM’s time. |
| Sponsor | 5b | Bill & Melinda Gates Foundation |
| Role of sponsor or funder | 5c | The funder did not have a role in this review. |
| **INTRODUCTION** | | |
| Rationale | 6 | Measles is a highly infectious, acute systemic viral infection, responsible for over 100,000 deaths annually, despite wide use of a safe and effective vaccine.^1^ Global coverage of the first dose of measles containing vaccine (MCV1) is estimated as 86%, with wide variation observed between and within countries.^2^ Introductions of the second dose (MCV2) has accelerated in the last decade, with 171 countries now including the vaccine in routine childhood vaccination schedules and approximately 69% of children receiving the second dose.^2^ Since 2000, an estimated 23.2 million deaths have been averted using the measles vaccine, and global measles deaths have declined by 73%.^3^ In 2016, PAHO declared measles eliminated from the whole Region of the Americas.^4^ Despite these achievements, the resurgence of measles threatens to undermine the progress made towards measles control. Global MCV1 coverage has remained stagnant for the last decade^5^ and MCV2 coverage – although increasing – is approximately 69%.^2,6^ In 2019, the United States reported 1,282 measles cases from 31 states, the greatest number of cases reported since 1992.^6,7^ Surveillance data from the World Health Organization (WHO) indicates that in 2019, confirmed measles cases occurred in 147 member states (i.e., countries) and all WHO regions experienced measles outbreaks.^8^ Countries in both Europe and the Americas lost their “measles elimination” status (with measles elimination defined as the absence of endemic measles virus transmission in a region or other defined geogra­phical area for ≥12 months, in the presence of a high-quality surveillance system during 2018 and 2019.^6,9^    High quality vaccination programs routinely rely on two sources of data to identify outbreaks and populations at highest risk: (1) vaccination coverage monitoring and (2) measles surveillance, which can be aggregate and/or case based. Serological surveillance for anti-measles IgG is potentially a more direct and timelier tool to identify susceptible populations and intervene prior to an outbreak.^10^ Serological surveillance, which allows the assessment of vaccine failure, can also be used to assess the effectiveness of vaccination programs, transmission dynamics and predict risk of future epidemics of VPDs.^11^    One of the challenges of serological surveillance is finding an accurate and feasible assay to measure anti-measles IgG concentrations for large quantities of samples. Plaque reduction neutralization testing (PRNT) is considered the “gold standard” because it is a functional antibody assay that measures the neutralization activity of anti-measles antibodies, regardless of isotype.^11-14^ Measles neutralizing antibodies reflect protective immunity, and therefore provide an important surrogate measure of vaccine effectiveness and population immunity through wild-type infection. Quantitative values from PRNT show good correlation with immune status and predict protection against infection and measles disease.^15^ However, there are several important disadvantages to using PRNT in serological studies. It is technically demanding, labor-intensive, and time-consuming resulting in low sample throughput. Furthermore, the procedure and interpretation of PRNT are variable and therefore difficult to standardize between laboratories.^15,16^ These factors limit the utility of PRNT in large-scale surveillance. Other serological techniques, such as antibody detection via enzyme immunosorbent assay (EIA), therefore play an important role in diagnostic case confirmation,^17^ and show promise for evaluating measles immunity within populations. EIAs are rapid, higher throughput, relatively inexpensive, and can be conducted in most laboratories with basic equipment using commercially available assays. However, EIAs measure all IgG isotype-specific epitopes of the target antigen/antibody, regardless of whether they are neutralizing or non-neutralizing.^18^ In addition, EIAs tend to be less sensitive than PRNT, especially with low antibody titers.^12,16,19-22^ This may be problematic for populations with poor antibody responses as a result of immunological immaturity, interference by passively acquired maternal antibodies, or waning antibody levels after prolonged periods since vaccination.^17^    An assessment of the accuracy of measles IgG EIAs compared to gold standard PRNTs is needed as serological surveillance is increasingly being used globally as a surveillance method for monitoring population immunity, with varying sample sources and methodologies. It is also increasingly important as vaccination coverage increases over time, and as countries achieve elimination, because the level of immunity in highly vaccinated populations is lower and EIA may not be sensitive enough to detect protective levels of immunity. Although direct comparisons of measles IgG EIAs versus PRNT have been periodically reported in the literature, such comparisons are often not the main objective of the analyses and results are not reported consistently. The objective of this systematic review is to assess the performance of measles IgG EIAs compared to PRNT for measuring seroprotection, seropositivity, and/or vaccine response. |
| Objectives | 7 | The aim of this review is to synthesize evidence to assess the performance of measles IgG EIAs for measuring serostatus (for purposes of measuring seroprotection, assuming a 120 mIU/mL cutoff^23^), seropositivity (assuming different EIA cutoffs), or vaccine response compared to PRNT. Specific objectives are to answer the following questions:   1. How accurate are EIAs for measles IgG for classifying serostatus (for purposes of measuring seroprotection, seropositivity, or responses to vaccine compared to PRNT? 2. How accurate are EIAs for measles IgG for quantifying antibody concentrations compared to PRNT? 3. What characteristics of the population, specimen, and/or assay are associated with poor performance of EIA compared to PRNT? Specifically: are there trends in underperformance by EIA characteristics; what are the risk factors for misclassification via EIA; and can strategies be identified to improve the accuracy of EIA compared to PRNT? 4. How does the estimated seroprevalence of measles IgG when using EIA compare to PRNT at the population level, where relevant?   Types of studies: Studies (of any study design) that report results from comparisons of any blood specimens (e.g., sera, whole blood, plasma) tested for measles IgG by EIA (commercial or in-house, including bead-based assays) and PRNT from the same source population will be included. We will distinguish if tests were conducted on the same specimen or just within the same population in data abstraction.  Population: All ages, with and without previous measles infection from all settings.    Intervention (index test): Enzyme immunoassay (EIA) for measles IgG.  Comparison (referent test): Plaque reduction neutralization test (PRNT) for measles.  Outcomes:  (1) EIA methods: antibody concentrations or the proportion of samples positive, equivocal, negative; in-house or commercial kit; type of EIA (indirect, sandwich, bead-based, direct); how equivocal results are handled; kit type/manufacturer; standardization methods; threshold of protection; limit of detection (upper and lower); reproducibility  (2) PRNT methods: antibody concentrations or the proportion of samples positive, equivocal, negative; how concentrations are reported (e.g., in-house or international units [IU]; if IU, which reference preparation was used and methods for conversion to IU); PRN standardization; threshold of protection;  (3) EIA vs PRNT performance: sensitivity; specificity; positive predictive value; negative predictive value; c-statistic; R^2^; kappa; percent agreement; other (to be defined as identified and needed during data abstraction) |
| **METHODS** |  |  |
| Eligibility criteria |  | Studies that report results from direct comparisons of blood specimens tested for measles IgG by EIA and PRNT from the same source population. The reference test will be PRNT; the index test will be any enzyme immunoassay (commercial or in-house, including bead-based assays). We will include peer-reviewed literature, appropriate grey literature, and unpublished results (if they meet quality criteria) reporting results from studies conducted in humans from 1947 through February 1, 2020. There will be no language restrictions unless the article cannot be translated.  The following studies will be excluded:  (1) Studies that do not compare EIA (measles IgG) to PRNT results (e.g., the study does not report the comparison, only one test was conducted for measles, or the tests were used to assess for a disease other than measles);  (2) Animal studies;  (3) Articles with no abstract or reviews and/or meta-analyses;  (4) Studies that measured IgA or IgM only;  (5) Basic science literature, such as vaccine development, that does not focus on comparing measles IgG by EIA against PRNT. |
| Information sources |  | (1) PubMed and EMBASE databases from the date of database creation through February 1, 2020;  (2) Reference lists of articles captured from PubMed and EMBASE databases and included in review;  (3) Subject matter experts to identify unpublished results and/or study authors for additional information as needed. |
| Search strategy |  | PubMed search:  ((((("Measles"[Text Word] OR "mmr"[Text Word]) OR "measles vaccine"[MeSH Terms]) OR "measles virus"[MeSH Terms]) OR "Measles"[MeSH Terms]) AND ((((((((("immunoenzyme techniques"[MeSH Terms] OR "immunoassay"[MeSH Terms]) OR ("enzyme linked"[All Fields] AND "immunosorbents"[MeSH Terms])) OR "enzyme-linked immunosorbent assay"[MeSH Terms]) OR "eia"[Text Word]) OR "ELISA"[Text Word]) OR "immunobead*"[Text Word]) OR "immunoenzyme*"[Text Word]) OR "enzyme immunoassay"[Text Word]) OR "enzyme linked immuno*"[Text Word])) AND ((((((("neutralization tests"[MeSH Terms] OR "viral plaque assay"[MeSH Terms]) OR "PRNT"[Text Word]) OR "PRN"[Text Word]) OR "PNT"[Text Word]) OR "gold standard"[Text Word]) OR "plaque reduction neutrali*"[Text Word]) OR "plaque neutrali*"[Text Word])    Embase search:  ('measles'/exp OR 'measles vaccine'/exp OR 'measles virus'/exp OR 'measles':ab,kw,ti,ok,lnk OR 'mmr*':ab,kw,ti,ok,lnk) AND ('enzyme immunoassay'/exp OR 'immunoassay'/exp OR 'enzyme linked immunosorbent assay'/exp OR 'elisa kit'/exp OR eia*:ab,kw,ti,ok,lnk OR elisa:ab,kw,ti,ok,lnk OR immunobead*:ab,kw,ti,ok,lnk OR immunoenzyme*:ab,kw,ti,ok,lnk OR 'enzyme immunoassay':ab,kw,ti,ok,lnk OR 'enzyme-linked immuno*':ab,kw,ti,ok,lnk) AND ('viral plaque assay'/exp OR 'serodiagnosis'/exp OR prnt:ab,kw,ti,ok,lnk OR prn:ab,kw,ti,ok,lnk OR pnt:ab,kw,ti,ok,lnk OR 'gold standard':ab,kw,ti,ok,lnk OR 'plaque reduction neutrali*':ab,kw,ti,ok,lnk OR 'plaque neutrali*':ab,kw,ti,ok,lnk)  Snowball inclusion:  Reference lists of articles included via title/abstract screening will be reviewed for relevant citations. |
| Study records: |  |  |
| Data management | 11a | Covidence will be used to manage records and data throughout the review; EndNote will be used as a secondary source for duplicate identification and full-text storage. |
| Selection process | 11b | Following a calibration exercise, two independent researchers will screen titles and abstracts for inclusion in full-text review using pre-qualified eligibility criteria. Full-text screening will be completed by two reviewers and inclusion in the final review will be based on the same inclusion criteria for title/abstract, the appropriateness of data for extraction, and quality assessment, as well as additional considerations where appropriate. Discrepancies will be reconciled between the two reviewers at each stage; if a consensus cannot be made, a third reviewer will be consulted.  We recognize that studies are heterogenous in many respects, particularly concerning the type of EIA (i.e, “kit”) used. Analyses will include stratification by EIA kits and/or cutoff used. There is no way to hypothesize *a priori* how many studies can be pooled and meta-analysis may not be possible. However, if possible, meta-analysis for pooled sensitivity and pooled specificity will be conducted. |
| Data collection process | 11c | A data extraction form created *a priori*, pilot-tested, and agreed upon by all authors of the systematic review will be used by two researchers. Authors of included studies will be contacted for clarification or additional information, if required. |
| Data items | 12 | The following are variables for which data will be extracted:  Study design/setting:  Specific objectives of the study; country in which study was conducted; disease setting (e.g., measles endemicity status and measles vaccine coverage in the country); age of the population from whom specimen samples were taken; population type (e.g., healthcare workers); previous measles infection and/or vaccination status of the population; specimen type (e.g., serum, plasma, whole blood, capillary vs. venous blood); number of samples overall; number of samples tested by both EIA and PRNT; blinding of laboratories to vaccination and disease status of specimens.  EIA methods:  Antibody concentrations or the proportion of samples positive, equivocal, negative; in-house or commercial kit; kit type (indirect, sandwich, bead-based, direct EIA); how equivocal results are handled; kit type/manufacturer; standardization methods; threshold of protection; limit of detection  PRNT methods:  Antibody concentrations or the proportion of samples positive, equivocal, negative; how concentrations are reported (e.g., in-house or international units [IU]; if IU, which reference preparation was used and methods for conversion to IU); PRN standardization; threshold of protection  EIA vs PRNT performance:  Sensitivity; specificity; positive predictive value; negative predictive value; c-statistic; R^2^; kappa; percent agreement; other (to be defined as identified and needed during data abstraction) |
| Outcomes and prioritization | 13 | Of the above listed data items, we define “outcomes” as those included in “EIA vs PRNT performance.” Prioritization will be given to sensitivity and specificity, measures of EIA accuracy, in order to pool these quantities for Bayesian meta-analysis if possible. |
| Risk of bias in individual studies | 14 | We will use the QUADAS-2 revised tool to assess risk of bias of individual studies. As recommended, we will score each item as “high risk of bias”, “low risk of bias”, or “unclear”.^24^ Information obtained from bias assessment may be used to exclude studies from inclusion or to exclude them just for sensitivity analyses. |
| Data synthesis |  |  |
|  | 15a | Each study will be quantitatively assessed for the reported accuracy with which EIAs can measure measles IgG compared to PRNTs. Priority measures assessed are sensitivity and specificity of the EIA, but others – such as positive predictive value, negative predictive value, kappa, and correlation – will also be extracted and summarized. |
|  | 15b | We anticipate considerable heterogeneity in the EIA kits used in included studies. Quantitative data will be synthesized in summary tables; average values, ranges, and individual measures will be reported. Furthermore, forest plots will be generated to display sensitivity and specificity. If possible, a Bayesian meta-analysis for pooled sensitivity and pooled specificity will be conducted and a hierarchical summary receiver operating characteristics (HSROC) curve will be plotted. |
|  | 15c | Subgroup analyses may be conducted as appropriate for specific populations or if studies report indeterminate assay results. |
|  | 15d | In addition to quantitative assessment, included studies will be qualitatively assessed for the reported extent to which EIAs are able to classify measles serostatus at the population level compared to PRNT. |
| Meta-bias(es) | 16 | Potential publication bias across studies will be assessed following recommendations by the Cochrane Collaboration Diagnostic Test Accuracy Working Group (e.g., Deeks’ test).^25-28^ In order to address language bias, we will not restrict our search to English-only articles. |
| Confidence in cumulative evidence | 17 | We will use the Grading of Recommendations Assessment, Development, and Evaluation (GRADE) approach, a transparent and systematic process for making judgements about quality of the evidence. |

# **Supplemental Table 2A: Studies evaluating EIA compared to PRNT (Medium quality)**

| Study | Country of sample collection | Elimination status at time of study | Time since elimination | Objective | N of samples tested | Age | Type of study subjects/samples | Eligibility criteria | EIA | EIA threshold | PRNT threshold | Subsample selected |
| --- | --- | --- | --- | --- | --- | --- | --- | --- | --- | --- | --- | --- |
| Bolotin 2019 | Canada | Eliminated | 15 yrs 10 months (November 2013- January 1998) to 16 yrs 5 m(May 2014 - January 1998) | Population-based seroprevalence study | 245 | 1-39yrs | Residual serum samples collected from a private diagnostic laboratory, which test samples from OPD and hospitalized patients. Patients born before 1970 were excluded. | Random sample or source unrelated to exposure or outcome | BioPlex 2200 | <0.13 AU/mL | >192 mIU/mL | All EIA negative and equivocal samples tested on PRNT. |
| Castro-Silva 2003 | Brazil | Eliminated | - | Population-based seroprevalence study | 57 | NR | Plasma samples obtained from HEMORIO bank. | Random sample or source unrelated to exposure or outcome | Enzygnost | NR | >120 mIU/mL | All EIA negative samples tested on PRNT. |
| Coughlin 2021^#^ | USA, Tajikistan | Eliminated (US)*; Endemic (Tajikistan) | NA (US); -(Tajikistan)) | Diagnostic accuracy | 516 | 6m - adults | Residual serum samples obtained routine case-based surveillance (US), early revaccination cohort (US) and a serosurvey (Tajikistan). | Random sample or source unrelated to exposure or outcome | In house MBA (MeV WVA_c_ ) | <153mIU/mL | ≥120 mIU/mL^b^ | All tested |
| Cremer 1985 | USA | Endemic | - | Diagnostic accuracy | 113 | NR | Serum samples selected ranging from low, middle and high EIA index. Emphasis was placed on samples with low EIA index (1 to <5). | Results from previous tests | In house EIA | <1.0 O.D. | ≥1:4^b^ | All samples with low EIA index tested on PRNT. |
| Erdman 1991 | USA | Endemic | - | Diagnostic accuracy | 32 | ≥15m- 18yrs | Serum samples collected pre, 3 weeks post and 8 post after MMR vaccination. | Selected based on vaccination status | In house EIA | <0.11 O.D. | Titer of ≥4 | All tested |
| Hesketh 1997 | United Kingdom | Endemic | - | Diagnostic accuracy | 101 | NR | Serum samples from a wide donor age range with known HI titers. | Results from previous tests | Gull; Human; Incstar; Measelisa II; Measelestat; Melotest; Platest; Enzygnost; Sigma | NR | NR | Samples with sufficient volume. |
| Latner 2020 | USA | Eliminated* | NA | Diagnostic accuracy | 146 | NR | Serum samples with known EIA results and available volume. | Results from previous tests and random sample or source unrelated to exposure or outcome | Diasorin;  Trinity;  Vidas;  Zeus;  BioPlex | <25.0 AU/ml, <13.5 AU/mL;  NR | ≥120 mIU/mL^b^,  ≥60 mIU/mL^b^,  ≥40mIU/mL ^b^,  ≥8 mIU/mL^b^; | All tested |
| Mancuso 2008 | USA | Endemic | - | Population-based seroprevalence study | 96 | >18yrs | Samples obtained from a study among US military recruits. | Recruited from community or healthcare setting, not related to measles infection or vaccination | Wampole | NR | ≥120 mIU/mL; 8mIU/mL | Random subset EIA negative and positive samples tested on PRNT. |
| Matson 1993 | USA | Endemic | - | Measles outbreak investigation and predict efficacy of measles booster dose | 43; 24 | ≤18yrs | Mix of samples obtained from school children in grades 6-12 before measles revaccination after a measles outbreak and 9-10 months after measles revaccination. | Exposure to measles outbreak | Measlestat | 0.0 to 0.099 absorbance | >1:900 | All EIA negative samples tested on PRNT. |
| Morris 2015 | USA | Eliminated | 11 yrs 5 m (June 2011- January 2000) to 11 yrs 10 m (October 2011- January 2000) | Diagnostic accuracy | 33 | 13-26yrs | Serum samples from perinatally HIV-infected adolescents and young adults with prior MMR vaccination. | Selected based on vaccination status | Vidas | >700 mIU/mL | ≥120 mIU/mL^b^ | Samples with sufficient volume. |
| Oliveira 1996 | Brazil | Endemic | - | Population-based seroprevalence study | 78 | 1-19yrs | Serum samples collected from students in schools in 3 district using probability sampling design. | Random sample or source unrelated to exposure or outcome | In house EIA | P-N ≥0.09 | ≥50 mIU/mL^b^ | All EIA negative samples tested on PRNT. |
| Tapia 2005 | Mali | Endemic | - | Persistence of vaccine-induced antibody | 24 | 9-10m | Serum samples obtained 3-5 weeks after measles vaccine receipt. | Selected based on vaccination status | In house EIA | <200 mIU/mL | ≥ 200 mIU/mL | All tested |
| Wangchuk 2019 | Bhutan | Eliminated | 1 year 2 months (March 2017-Jan 2016) to 1 year 4 months (Apr 2017 - Jan 2016) | Population-based seroprevalence study | 58 | 5-11yrs | Serum samples obtained from a randomized multi-stage cluster serosurvey. | Random sample or source unrelated to exposure or outcome | Enzygnost | <150 mIU/mL | ≥120 mIU/mL | Subset of EIA negative samples with 2 doses of MRCV were tested on PRNT. |
| Weigle 1984 | USA | Endemic | - | Diagnostic accuracy | 11;22 | >18yrs; ≥15m- 18yrs | Age >18 yrs: Serum obtained from blood bank of adults born before 1954 (before measles vaccine introduction) and working in pediatric OPD;  Age ≥15m- 18yrs: Serum obtained from MMR vaccinated children. | Selected based on vaccination status | In house EIA | ≤0.062 O.D. | ≥120 mIU/mL^b^ | Samples with sufficient volume. |

EIA, Enzyme immunoassay. HIV, Human Immunodeficiency virus. MRCV, Measles and rubella- containing vaccine. MMR, Measles, mumps and rubella vaccine. MBA, Multiplex Bead Assay. MeV WVA_c_, Commercially produced whole-virus antigen. mIU/mL, milli-international units per milliliter. m, months. NA, Not available. NR, Not reported. O.D., Optical density. yrs, years. PRNT, Plaque Reduction Neutralization Test. USA, United States of America.^a^ EIA thresholds reported did not use or did not explicitly report to use manufacturers recommendation.^b^ Reported to use methods other than those described in Albrecht et. al. 1981 or did not describe methodology. ^#^Coughlin 2021 was classified as a high quality study however the result reported for comparison of PRNT with In house MBA (MeV WVAc) and so the results are reported here. *Elimination status was assumed using publication year as date of specimen collection was not reported.

#

# **Supplemental Table 2B: Studies evaluating EIA compared to PRNT (Low quality)**

| Study | Country of sample collection | Elimination status at time of study | Time since elimination | Objective | N of samples tested | Age | Type of study subjects/samples | Eligibility criteria | EIA | EIA threshold | PRNT threshold | Subsample selected |
| --- | --- | --- | --- | --- | --- | --- | --- | --- | --- | --- | --- | --- |
| Al-Mazrou 2002 | Kingdom of Saudi Arabia | Endemic | - | Population-based seroprevalence study | 150 | 6m-17yrs | Serum samples obtained from a national seroprevalence study after MMR vaccination campaign. | Random sample or source unrelated to exposure or outcome. | Enzygnost | <0.1 O.D. | ≥40 mIU/mL | All EIA negative and equivocal samples tested on PRNT. |
| Al-Mazrou 2005 | Kingdom of Saudi Arabia | Endemic | - | Population-based seroprevalence study | 109 | 6m- 17yrs | Serum samples obtained from a national seroprevalence study after a MMR vaccination campaign. | Random sample or source unrelated to exposure or outcome | Enzygnost | <150 mIU/mL | ≥1:2^b^ | All EIA negative and equivocal samples tested on PRNT. |
| Coughlin 2021^#^ | USA, Tajikistan | Eliminated (US)*; Endemic (Tajikistan); Endemic (Bangladesh) | NA (US); - (Tajikistan); -(Bangladesh) | Diagnostic accuracy | 140, 212, 516 | 6m - adults | Residual serum samples obtained routine case-based surveillance (US), early revaccination cohort (US) and a serosurvey (Tajikistan) | Random sample or source unrelated to exposure or outcome. | Enzygnost;  Zeus | NR | ≥120 mIU/mL^b^ | All tested |
| Job 1991 | Haiti | Endemic | - | Evaluate primary response to vaccination | NR | 6-11m | Serum samples obtained from infants living in a periurban slum before vaccination, 2 months and 12 months after measles vaccination. | Selected based on vaccination status | Measlestat | <200 mIU/mL | ≥200 mIU/mL | Samples with sufficient volume |
| Kang 2017 | Republic of Korea | Eliminated | 8 yrs (January 2014 - January 2006) to 9 yrs (December 2014 -January 2006) | Population-based seroprevalence study | 480 | 10- 50yrs | Residual serum specimens obtained from a private diagnostic laboratory and National Health and Nutrition Examination Survey (KNHANES VI-1^st^). Excluded samples referred for diagnosis of measles, mumps, rubella or HIV. | Random sample or source unrelated to exposure or outcome. | Enzygnost | <0.1 O.D. | ≥120 mIU/mL^b^ | Random subset of EIA samples tested on PRNT. |
| Kidokoro 2002 | Japan | Endemic | - | Diagnostic accuracy | NR | NR | Serum samples obtained from cells grown in Minimal Eagle’s medium. | Selected based on vaccination status | Enzygnost | NR | NA** | All tested |
| Ng 2020 | Singapore | Eliminated | 2 yrs (January 2015-2017( to 2 yrs 9m (January 2015-September 2018) | Population-based seroprevalence study | 145 | 1-17yrs | Residual serum samples obtained from from diagnostic laboratories of pediatric departments of 2 public hospitals. Excluded non-citizens/permanent residents, immunocompromised, undergoing immuno-suppressive therapy etc. | Recruited from community or healthcare setting, not related to measles infection or vaccination. | Enzygnost | <0.1 O.D. | NR | All EIA negative and a random subset of EIA equivocal samples tested on PRNT. |
| Pabst 1999 | Canada | Endemic | - | Evaluate primary response to vaccination | 300 | 6m | Serum samples collected from infants before and after measles vaccination born to mothers with history of measles infection and vaccinated mothers. | Selected based on vaccination status. | Enzygnost | <0.200 O.D. | NR | Subset of EIA samples tested on PRNT. |
| Pourabbas 2008 | Iran | Endemic | - | Population-based seroprevalence study | 120 | 6-10, 11-15, 16-20 & 21-26yrs | Serum samples collected from general population using random sampling method. | Random sample or source unrelated to exposure or outcome | IBL | < 8000 mIU/mL | >1:120^b^ | All EIA equivocal or indeterminate tested on PRNT. |
| Siennicka 2014 | Poland | Endemic | - | Diagnostic accuracy | 5 | NR | NR | NR | Enzygnost | < 0.100 O.D. | NR | All tested |
| Tischer, Gassner 2007 | Switzerland | Endemic | - | Diagnostic accuracy | 101 | 13-15yrs | Serum samples obtained from high school students with known measles vaccination status. | Random sample or source unrelated to exposure or outcome. | Enzygnost; NR | <150 mIU/mL;  <400 mIU/mL | ≥40 ± 20 mIU/mL^b^ | All EIA negative and equivocal samples tested on PRNT. |

EIA, Enzyme immunoassay. HIV, Human Immunodeficiency virus. MMR, Measles, mumps and rubella vaccine. m, months. PRNT, NA, Not available. NR, Not reported. O.D., Optical density. Plaque Reduction Neutralization Test. yrs, years. ^a^EIA thresholds reported did not use or did not explicitly report to use manufacturers recommendation. ^b^Reported to use methods other than those described in Albrecht et. al. 1981 or did not describe methodology. *Elimination status was assumed using publication year as date of specimen collection was not reported. **Authors reported NT but methods were comparable to PRNT. Authors sought to identify a threshold, and one was not pre-specified. ^#^Coughlin 2021 was classified as a high quality study however the results reported for comparison of PRNT with Enzynost and Zeus kits were low quality and so the results are reported here.

**Supplementary Table 3: Median (IQR) diagnostic accuracy of EIA compared to PRNT by assay type and study quality.**

| Assay/kits | Study quality | Number of comparisons assessed* | Median [IQR] sensitivity | Median [IQR] specificity | N studies with both sensitivity and specificity > 90.0% |
| --- | --- | --- | --- | --- | --- |
| All, Enzygnost | High | 12 | 92.1 [82.3, 95.7] | 96.9 [93.0, 100.0] | 6 |
| All, Other EIA | High | 19 | 90.6 [86.6, 95.2] | 100.0 [88.7, 100.0] | 6 |
| All | Medium | 22 | 93.1 [86.8, 100.0] | 85.7 [79.6, 100.0] | 1 |
| Enzygnost | Medium | 3 | 92.0 [92.0, 92.0] | 100.0 [86.0, 100.0] | 0 |
| Vidas | High | 3 | 90.0 [82.5, 90.5] | 100.0 [97.5,100.0] | 1 |
| Vidas | Medium | 2 | 77.1 [72.1, 82.2] | 89.7 [88.0, 91.3] | 0 |
| Diasorin | Medium | 2 | 88.7 [88.0, 89.5] | 77.3 [76.1, 78.4] | 0 |
| MBA | High | 6 | 95.0 [89.8, 95.0] | 84.0 [80.5, 96.5] | 1 |
| MBA | Medium | 3 | 90.0 (87.6, 95.0) | 81.8 [57.9, 82.4] | 0 |
| In house EIA | High | 2 | 99.0 [99.0, 99.0] | 100.0 [100.0, 100.0] | 2 |
| In house EIA | Medium | 8 | 100.0 [91.0, 100.0] | 100 [95.0, 100.0] | 1 |

EIA, enzyme immunoassay. IQR, interquartile range. MBA, multiplexed bead-based assays. Reporting assays/kits with at least 3 EIA-PRNT comparisons assessed. *Point estimates which were not estimable, not reported or with low PRNT threshold (eg., 8mIU/mL) were excluded.

# **Supplementary Table 4: Diagnostic accuracy measures reported in medium quality studies**

| Study | N of samples tested | Age | EIA | EIA threshold | PRNT threshold | Subsample selected | EIA equivocal | TP | FP | FN | TN | Sensitivity  % (95%CI) | Specificity % (95%CI) | PPV % (95%CI) | NPV % (95%CI) | Kappa statistic | Correlation |
| --- | --- | --- | --- | --- | --- | --- | --- | --- | --- | --- | --- | --- | --- | --- | --- | --- | --- |
| Latner 2020 | 146 | NR | Diasorin | <25.0 AU/ml | ≥120 mIU/mL^b^ | All tested | NR | NR | NR | NR | NR | 87.2 (79.2-93.0)^d^ | 79.6 (64.7-90.2)^d^ | NR | NR | NR | NR |
|  |  |  |  |  | ≥60 mIU/mL^b^ | All tested | NR | NR | NR | NR | NR | 82.2 (74.1-88.6)^d^ | 96.0 (81.7-99.9)^d^ | NR | NR | NR | NR |
|  |  |  |  |  | ≥40 mIU/mL^b^ | All tested | NR | NR | NR | NR | NR | 81.0 (72.9-87.6)^d^ | 100.0 (86.3-100.0)^d^ | NR | NR | NR | NR |
|  |  |  |  |  | ≥8 mIU/mL^b^ | All tested | NR | NR | NR | NR | NR | 77.8 (69.5-84.7)^d^ | 100.0 (83.2-100.0)^d^ | NR | NR | NR | NR |
| Latner 2020 | 146 | NR | Diasorin | <13.5 AU/ml | ≥120 mIU/mL^b^ | All tested | NR | NR | NR | NR | NR | 90.2 (82.7-95.2)^d^ | 75.0 (59.7-86.8)^d^ | NR | NR | NR | NR |
|  |  |  |  |  | ≥60 mIU/mL^b^ | All tested | NR | NR | NR | NR | NR | 85.6 (77.9-91.4)^d^ | 92.9 (76.5-99.1)^d^ | NR | NR | NR | NR |
|  |  |  |  |  | ≥40 mIU/mL^b^ | All tested | NR | NR | NR | NR | NR | 84.3 (76.6-90.2)^d^ | 96.0 (79.7-99.9)^d^ | NR | NR | NR | NR |
|  |  |  |  |  | ≥8 mIU/mL^b^ | All tested | NR | NR | NR | NR | NR | 81.8 (73.9-88.1)^d^ | 100.0 (83.2-100.0)^d^ | NR | NR | NR | NR |
| Hesketh 1997 | 101 | NR | Gull | NR | NR | Samples with sufficient volume | NR | NR | NR | NR | NR | 94.0^d^ | 73.0^d^ | NR | NR | 0.58 | NR |
|  |  |  | Human | NR | NR | Samples with sufficient volume | NR | NR | NR | NR | NR | 97.0^d^ | 47.0^d^ | NR | NR | 0.40 | NR |
|  |  |  | Incstar | NR | NR | Samples with sufficient volume | NR | NR | NR | NR | NR | 93.0^d^ | 71.0^d^ | NR | NR | 0.53 | NR |
|  |  |  | Measelisa II | NR | NR | Samples with sufficient volume | NR | NR | NR | NR | NR | 70.0^d^ | 93.0^d^ | NR | NR | 0.34 | NR |
|  |  |  | Measlestat | NR | NR | Samples with sufficient volume | NR | NR | NR | NR | NR | 95.0^d^ | 50.0^d^ | NR | NR | 0.42 | NR |
|  |  |  | Melotest | NR | NR | Samples with sufficient volume | NR | NR | NR | NR | NR | 92.0^d^ | 73.0^d^ | NR | NR | 0.59 | NR |
|  |  |  | Platest | NR | NR | Samples with sufficient volume. | NR | NR | NR | NR | NR | 88.0^d^ | 57.0^d^ | NR | NR | 0.32 | NR |
|  |  |  | Enzygnost | NR | NR | Samples with sufficient volume. | NR | NR | NR | NR | NR | 92.0^d^ | 72.0^d^ | NR | NR | 0.47 | NR |
| Castro-Silva 2003 | 57 | NR | Enzygnost | NR | ≥120 mIU/mL^b^ | All EIA negative samples tested on PRNT. | NR | 0 | 0 | 5 | 52 | NE | 100.0 (93.2-100.0)^c^ | NE | 91.2 (80.7-97.1)^c^ | NR | NR |
| Wangchuk 2019 | 58 | 5-11yrs | Enzygnost | <150 mIU/mL | ≥120 mIU/mL | Subset of EIA negative samples with 2 doses of MRCV were tested on PRNT. | NR | 0 | 0 | 12 | 46 | NE | 100.0 (92.3-100.0)^c^ | NE | 79.3 (66.6-88.8)^c^ | NR | NR |
| Hesketh 1997 | 101 | NR | Sigma | NR | NR | Samples with sufficient volume | NR | NR | NR | NR | NR | 92.0^d^ | 73.0^d^ | NR | NR | 0.56 | NR |
| Latner 2020 | 146 | NR | Trinity | NR | ≥120 mIU/mL^b^ | All tested | NR | NR | NR | NR | NR | 93.1 (86.4-97.2)^d^ | 79.6 (64.7-90.2)^d^ | NR | NR | NR | NR |
|  |  |  |  |  | ≥60 mIU/mL^b^ | All tested | NR | NR | NR | NR | NR | 87.3 (79.9-92.7)^d^ | 96.4 (81.7-99.9)^d^ | NR | NR | NR | NR |
|  |  |  |  |  | ≥40 mIU/mL^b^ | All tested | NR | NR | NR | NR | NR | 88.2 (80.6-93.6) | 100.0 (86.3-100.0)^d^ | NR | NR | NR | NR |
|  |  |  |  |  | ≥8 mIU/mL^b^ | All tested | NR | NR | NR | NR | NR | 38.9 (30.3-48.0)^d^ | 100.0 (83.2-100.0)^d^ | NR | NR | NR | NR |
| Morris 2015 | 33 | 13-26yrs | Vidas | >700 mIU/mL | ≥120 mIU/mL^b^ | Samples with sufficient volume | NR | 4 | 2 | 2 | 26 | 66.7 (22.3-95.7)^c^ | 92.9 (76.5-99.1)^c^ | 66.7 (22.3-95.7)^c^ | 92.9 (76.5-99.1)^c^ | 0.59 | NR |
| Latner 2020 | 146 | NR | Vidas | NR | ≥120 mIU/mL^b^ | All tested | NR | NR | NR | NR | NR | 87.2 (79.2-93.0)^d^ | 86.4 (72.7-94.8)^d^ | NR | NR | NR | NR |
|  |  |  |  |  | ≥60 mIU/mL^b^ | All tested | NR | NR | NR | NR | NR | 80.5 (72.2-87.2)^d^ | 100.0 (87.7-100.0)^d^ | NR | NR | NR | NR |
|  |  |  |  |  | ≥40 mIU/mL^b^ | All tested | NR | NR | NR | NR | NR | 78.5 (70.1-85.5)^d^ | 100.0 (86.3-100.0)^d^ | NR | NR | NR | NR |
|  |  |  |  |  | ≥8 mIU/mL^b^ | All tested | NR | NR | NR | NR | NR | 75.4 (66.9-82.6)^d^ | 100.0 (83.2-100.0)^d^ | NR | NR | NR | NR |
| Latner 2020 | 146 | NR | Zeus | NR | ≥120 mIU/mL^b^ | All tested | NR | NR | NR | NR | NR | 96.1 (90.3-98.9)^d^ | 70.5 (54.8-83.2)^d^ | NR | NR | NR | NR |
|  |  |  |  |  | ≥60 mIU/mL^b^ | All tested | NR | NR | NR | NR | NR | 92.4 (86.0-96.5)^d^ | 92.9 (76.5-99.1)^d^ | NR | NR | NR | NR |
|  |  |  |  |  | ≥40 mIU/mL^b^ | All tested | NR | NR | NR | NR | NR | 91.7 (85.3-95.9)^d^ | 100.0 (86.3-100.0)^d^ | NR | NR | NR | NR |
|  |  |  |  |  | ≥8 mIU/mL^b^ | All tested | NR | NR | NR | NR | NR | 88.1 (81.1-93.2)^d^ | 100.0 (83.2-100.0)^d^ | NR | NR | NR | NR |
| Mancuso 2008 | 96 | >18yrs | Wampole | NR | ≥120 mIU/mL | Random subset EIA negative and positive samples tested on PRNT. | NR | 47 | 1 | 42 | 6 | 52.8 (41.9-63.5)^c^ | 85.7 (42.1-99.6)^c^ | 97.9 (88.9-99.9)^c^ | 12.5 (4.7-25.2)^c^ | NR | NR |
|  |  |  |  |  | ≥8 mIU/mL | Random subset EIA negative and positive samples tested on PRNT. | NR | 47 | 1 | 45 | 3 | 51.1 (40.4-61.7)^c^ | 75.0 (19.4-99.4)^c^ | 97.9 (88.9-99.9)^c^ | 6.3 (1.3-17.2)^c^ | NR | NR |
| Matson 1993 | 43 | ≤18yrs | Measlestat | 0.0 to 0.099 absorbance | >1:900 | All EIA negative samples tested on PRNT. | NR | 0 | 0 | 0 | 43 | NE | 100.0 (91.8-100.0)^c^ | NE | 100.0 (91.8-100.0)^c^ | NR | 0.80^e^ |
|  | 24 | ≤18yrs | Measlestat | 0.0 to 0.099 absorbance | >1:900 | All EIA negative samples tested on PRNT. | NR | 5 | 4 | 0 | 15 | 100.0 (47.8-100.0)^c^ | 78.9 (54.4-93.9)^c^ | 55.6 (21.2-86.3)^c^ | 100.0 (78.2-100.0)^c^ | NR | 0.80^e^ |
| Bolotin 2019 | 245 | 1-39yrs | BioPlex 2200 | <0.13 AU/mL^a^ | >192 mIU/mL | All EIA negative and equivocal samples tested on PRNT. | Grouped with positives | 151 | 62 | 0 | 32 | 100.0 (97.6-100.0)^c^ | 34.0 (24.6-44.5)^c^ | 70.9 (64.3-76.9)^c^ | 100.0 (89.1-100.0)^c^ | NR | NR |
| Latner 2020 | 146 | NR | BioPlex 2200 | NR | ≥120 mIU/mL^b^ | All tested | NR | NR | NR | NR | NR | 85.2 (76.7-91.4)^d^ | 81.8 (67.3-91.8)^d^ | NR | NR | NR | NR |
|  |  |  |  |  | ≥60 mIU/mL^b^ | All tested | NR | NR | NR | NR | NR | 79.5 (71.0-86.3)^d^ | 96.4 (81.7-99.9)^d^ | NR | NR | NR | NR |
|  |  |  |  |  | ≥40 mIU/mL^b^ | All tested | NR | NR | NR | NR | NR | 78.3 (69.9-85.3)^d^ | 100.0 (86.3-100.0)^d^ | NR | NR | NR | NR |
|  |  |  |  |  | ≥8 mIU/mL^b^ | All tested | NR | NR | NR | NR | NR | 75.2 (66.7-82.4) | 100.0 (83.2-100.0) | NR | NR | NR | NR |
| Weigle 1984 | 11 | > 18yrs | In house EIA | ≤0.062 O.D. | ≥1:8 | Samples with sufficient volume. | NR | 11 | 0 | 0 | 0 | 100.0 (71.5-100.0)^c^ | NE | 100.0 (72.0-100.0)^c^ | NE | NR | NR |
|  | 22 | ≥15m- 18yrs | In house EIA | ≤0.062 O.D. | ≥1:8 | Samples with sufficient volume. | NR | 17 | 0 | 0 | 5 | 100.0 (80.5-100.0)^c^ | 100.0 (47.8-100.0) | 100.0 (80.5-100.0)^c^ | 100.0 (47.8-100.0) | NR | NR |
| Cremer 1985 | 113 | NR | In house EIA | <1.0 O.D. | ≥1:4^b^ | All tested | NR | 83 | 6 | 0 | 24 | 100.0 (95.7-100.0)^c^ | 80.0 (61.4-92.3)^c^ | 93.3 (85.9-97.5) | 100.0 (85.8-100.0) | NR | NR |
| Erdman 1991 | 32 | ≥15m- 18yrs  (pre MMR vaccination) | In house EIA | <0.11 O.D. | <4 | All tested | NR | 0 | 0 | 0 | 32 | NE | 100.0 (89.1-100.0)^c^ | NE | 100.0 (89.1-100.0)^c^ | NR | NR |
|  |  | ≥15m- 18yrs  (3 weeks post MMR vaccination) | In house EIA | <0.11 O.D. | <4 | All tested | NR | 32 | 0 | 0 | 0 | 100.0 (89.1-100.0)^c^ | NE | 100.0 (89.0-100.0)^c^ | NE | NR | NR |
|  |  | ≥15m- 18yrs  (8 months post MMR) | In house EIA | <0.11 O.D. | <4 | All tested | NR | 19 | 0 | 13 | 0 | 59.4 (40.6-76.3)^c^ | NE | 100 (82-100)^c^ | 0.0 (0.0-25.0)^c^ | NR | NR |
| Oliveira 1996 | 78 | 1-19yrs | In house EIA | P-N ≥0.09 | ≥50 mIU/mL^b^ | All EIA negative samples tested on PRNT. | NR | 0 | 0 | 68 | 10 | NE | 100.0 (69.2-100.0)^c^ | NE | 12.8 (6.3-22.3)^c^ | NR | NR |
| Tapia 2005 | 148 | 9-10m | In house EIA | <200 mIU/mL | ≥ 200 mIU/mL | All tested | NR | 33 | 0 | 5 | 110 | 86.8 (71.9-95.6) ^c^ | 100 (96.7-100.0) ^c^ | 100 (89.4-100.0)^c^ | 95.7 (90.1-98.6)^c^ | NR | 0.93 |
| Coughlin 2021* | 516 | 6 m - adults | In house MBA  (MeV WVA_c_) | <153 mIU/mL | ≥120 mIU/mL^b^ | All tested | NR | NR | NR | NR | NR | 98.0 (95.8-99.6)^d^ | 83.0 (78.3-87.6)^d^ | NR | NR | NR | NR |

EIA, Enzyme immunoassay. PRNT, Plaque Reduction Neutralization Test. CI, confidence interval. PPV, Positive Predictive Value. NPV, Negative Predictive Value. TP, True positives. FN, False negatives. FP, False positives. TN, True negatives. m, months. yrs, years. NR, Not reported.NE, Not estimable. O.D., Optical density. BA, Multiplex Bead Assay. MeV WVA_c_, Commercially produced whole-virus antigen. ^a^EIA thresholds reported did not use or did not explicitly report to use manufacturers recommendation. ^b^Reported to use methods other than those described in Albrecht et. al. 1981 or did not describe methodology. ^c^Estimates presented were not reported by authors but calculated using data reported. ^d^Estimates presented are reported by authors. Estimates could not be re-calculated owing to lack of data. ^e^Authors reported the overall correlation of 0.80 for all 67 samples in this study. *Coughlin 2021 was classified as a high quality study however the result reported for comparison of PRNT with In house MBA (MeV WVAc) and so the results are reported here.

**Supplementary Figure 1: Summary of Quality Assessment of Diagnostic Accuracy Studies (QUADAS-2) (revised tool) results.**

#
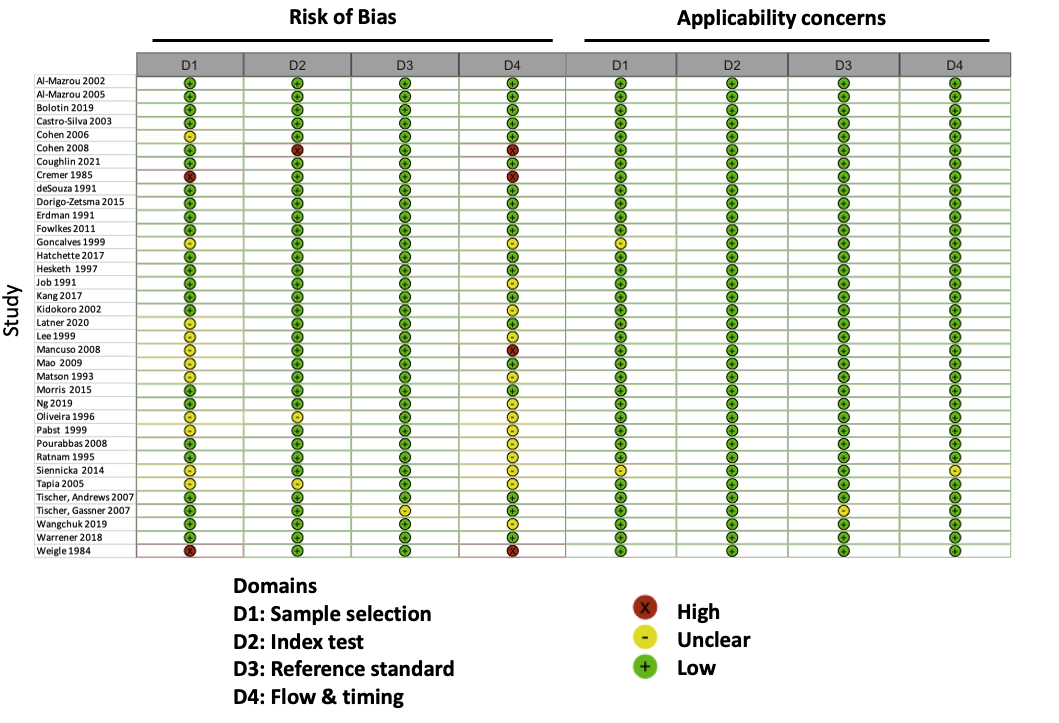


D, Domain.

# **Supplementary Figure 2: HSROC curves for measles EIA acompared to PRNT for high quality studies evaluating Siemens Enzygnost EIA kits.**

#

| **Diagnostic Accuracy** | **Estimate % (95% CI)** |
| --- | --- |
| Sensitivity | 91.6 (80.7-96.6) |
| Specificity | 96.0 (90.9-98.3) |

HSROC, hierarchical summary receiver operating characteristic.

**Supplementary Figure 3: Diagnostic accuracy of EIA compared to PRN reported in medium quality studies.**


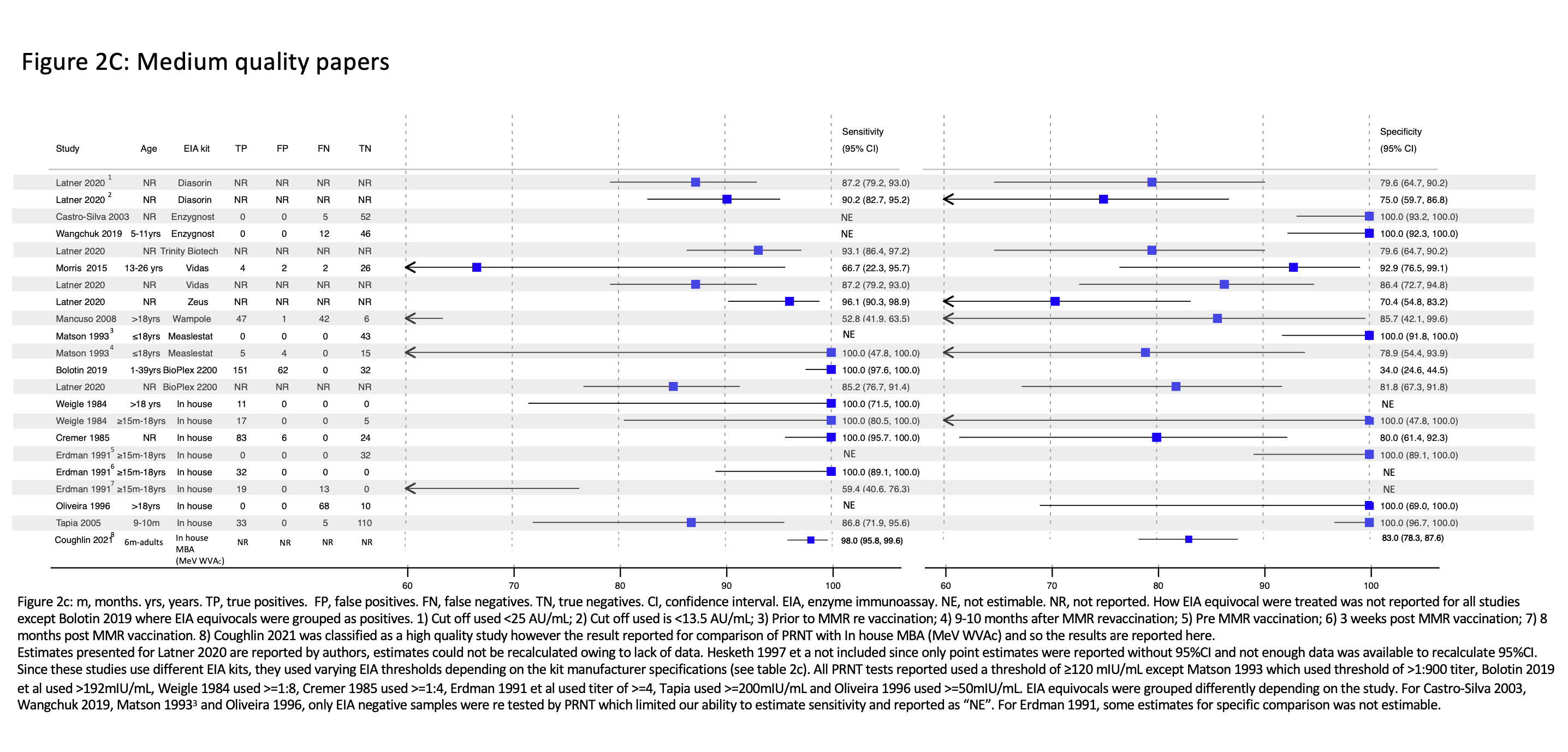


% sensitivity and specificity presented. m, months. yrs, years. TP, true positives. FP, false positives. FN, false negatives. TN, true negatives. CI, confidence interval. EIA, enzyme immunoassay. NE, not estimable. NR, not reported. How EIA equivocal were treated was not reported for all studies except Bolotin 2019 where EIA equivocals were grouped as positives. 1) Cut off used <25 AU/mL; 2) Cut off used is <13.5 AU/mL; 3) Prior to MMR re vaccination; 4) 9-10 months after MMR revaccination; 5) Pre MMR vaccination; 6) 3 weeks post MMR vaccination; 7) 8 months post MMR vaccination. 8) Coughlin 2021 was classified as a high quality study however the result reported for comparison of PRNT with In house MBA (MeV WVAc) and so the results are reported here. Estimates presented for Latner 2020 are reported by authors, estimates could not be recalculated owing to lack of data. Hesketh 1997 et a not included since only point estimates were reported without 95%CI and not enough data was available to recalculate 95%CI. Since these studies use different EIA kits, they used varying EIA thresholds depending on the kit manufacturer specifications. All PRNT tests reported used a threshold of ≥120 mIU/mL except Matson 1993 which used threshold of >1:900 titer, Bolotin 2019 et al used >192mIU/mL, Weigle 1984 used >=1:8, Cremer 1985 used >=1:4, Erdman 1991 et al used titer of >=4, Tapia used >=200mIU/mL and Oliveira 1996 used >=50mIU/mL. EIA equivocals were grouped differently depending on the study. For Castro-Silva 2003, Wangchuk 2019, Matson 1993^3^ and Oliveira 1996, only EIA negative samples were re tested by PRNT which limited our ability to estimate sensitivity and reported as “NE”. For Erdman 1991, some estimates for specific comparison was not estimable.

**Supplementary Figure 4: Diagnostic accuracy of EIA assays compared to PRN by assay type**


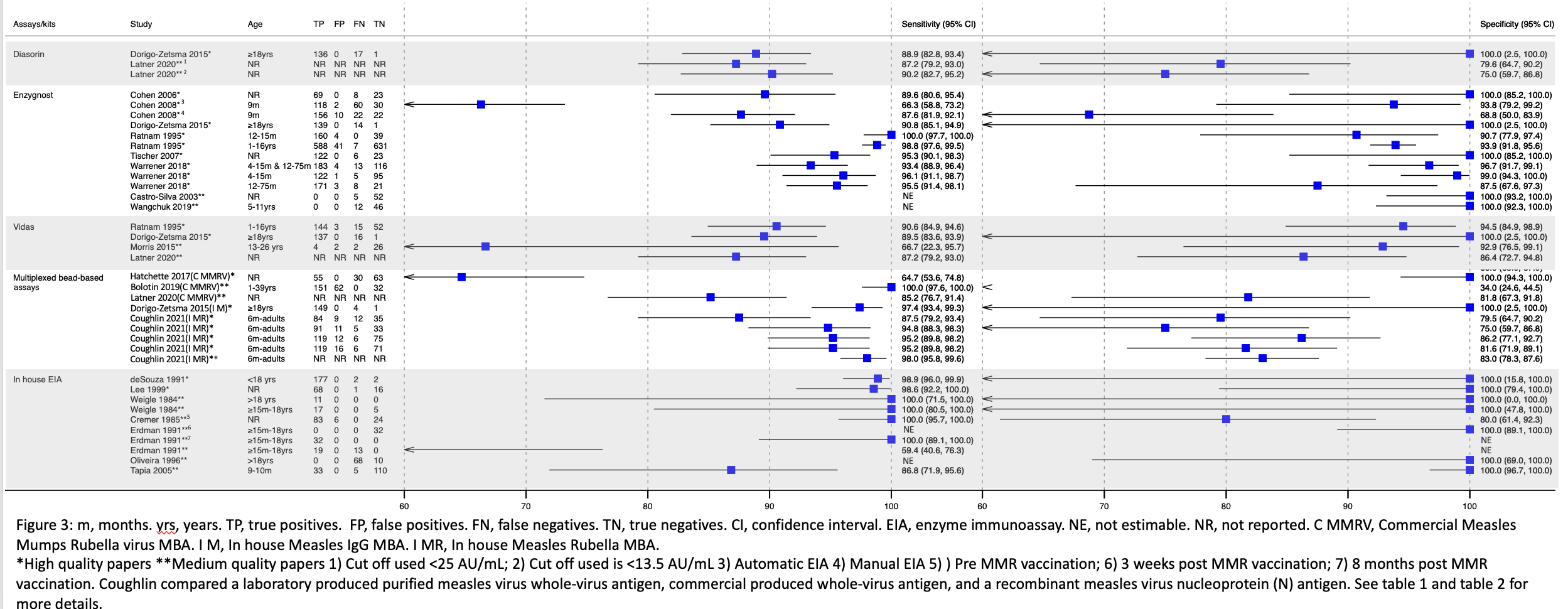


% sensitivity and specificity presented. m, months. yrs, years. TP, true positives. FP, false positives. FN, false negatives. TN, true negatives. CI, confidence interval. EIA, enzyme immunoassay. NE, not estimable. NR, not reported. C MMRV, Commercial Measles Mumps Rubella virus MBA. I M, In house Measles IgG MBA. I MR, In house Measles Rubella MBA.*High quality papers **Medium quality papers 1) Cut off used <25 AU/mL; 2) Cut off used is <13.5 AU/mL 3) Automatic EIA 4) Manual EIA 5) ) Pre MMR vaccination; 6) 3 weeks post MMR vaccination; 7) 8 months post MMR vaccination. Coughlin compared a laboratory produced purified measles virus whole-virus antigen, commercial produced whole-virus antigen, and a recombinant measles virus nucleoprotein (N) antigen.

**Supplementary Figure 5: Diagnostic accuracy of EIA compared to PRNT when EIA equivocals are re-classified, compared to results reported in high quality studies**


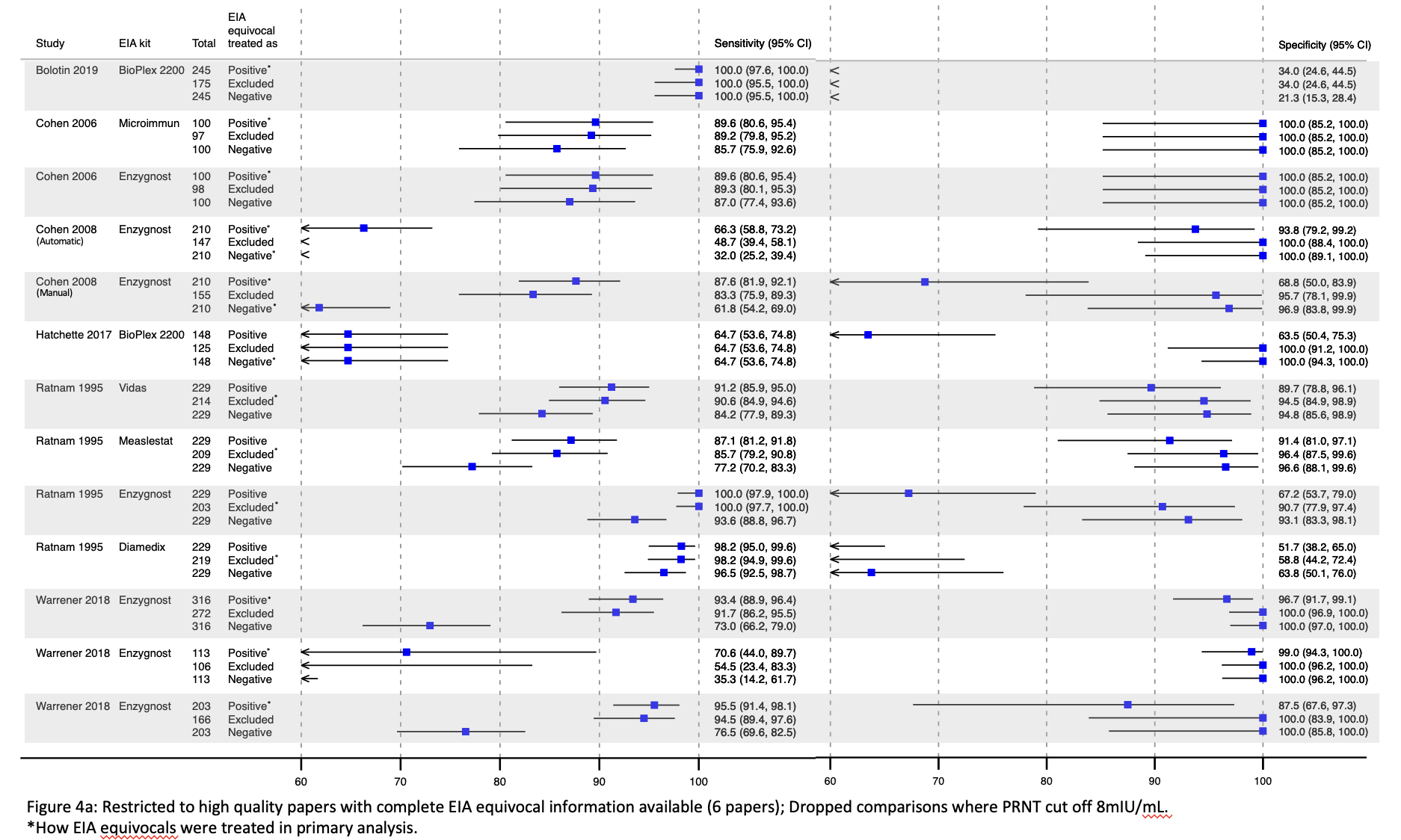


Restricted to high quality papers with complete EIA equivocal information available (6 papers); Dropped comparisons where PRNT cut off 8mIU/mL. *How EIA equivocals were treated in primary analysis.

**REFERENCES**

1. Moss WJ. Measles. *Lancet* 2017; **390**(10111): 2490-502.

2. World Health Organization. Immunization coverage. 2019. <https://www.who.int/news-room/fact-sheets/detail/immunization-coverage>. Accessed February 12, 2020.

3. World Health Organization. Measles. 2019. <https://www.who.int/news-room/fact-sheets/detail/measles>. Accessed January 3, 2020.

4. Pan American Health Organization. Region of the Americas is Declared Free of Measles. Immunization Newsletter. 2016;XXXVIII(3). <https://www.paho.org/en/file/52626/download?token=-9JbhBat>.

5. World Health Organization. Measles cases spike globally due to gaps in vaccination coverage. 2018. <https://www.who.int/news-room/detail/29-11-2018-measles-cases-spike-globally-due-to-gaps-in-vaccination-coverage>. Accessed January 28, 2020.

6. Patel M, Lee AD, Clemmons NS, et al. National Update on Measles Cases and Outbreaks - United States, January 1-October 1, 2019. *MMWR Morb Mortal Wkly Rep* 2019; **68**(40): 893-6.

7. Centers for Disease Control and Prevention. Measles (rubeola) cases and outbreaks. 2020. <https://www.cdc.gov/measles/cases-outbreaks.html>. Accessed January 24, 2020.

8. World Health Organization. Measles and rubella surveillance data. 2020. <https://www.who.int/immunization/monitoring_surveillance/burden/vpd/surveillance_type/active/measles_monthlydata/en/>. Accessed February 12, 2020.

9. World Health Organization Regional Office for Europe. European region loses ground in effort to eliminate measles. 2019. <http://www.euro.who.int/en/media-centre/sections/press-releases/2019/european-region-loses-ground-in-effort-to-eliminate-measles>. Accessed January 24, 2020.

10. Lessler J, Metcalf CJ, Cutts FT, Grenfell BT. Impact on Epidemic Measles of Vaccination Campaigns Triggered by Disease Outbreaks or Serosurveys: A Modeling Study. *PLoS Med* 2016; **13**(10): e1002144.

11. Cutts FT, Hanson M. Seroepidemiology: an underused tool for designing and monitoring vaccination programmes in low- and middle-income countries. *Trop Med Int Health* 2016; **21**(9): 1086-98.

12. Albrecht P, Herrmann K, Burns GR. Role of virus strain in conventional and enhanced measles plaque neutralization test. *J Virol Methods* 1981; **3**(5): 251-60.

13. Griffin DE. Measles virus. In: Knipe DM, Howley PM, eds. Fields virology. Philadelphia: Lippincott Williams & Wilkins; 2001: 1401-41.

14. Science M, Savage R, Severini A, et al. Measles Antibody Levels in Young Infants. *Pediatrics* 2019; **144**(6).

15. Cohen BJ, Audet S, Andrews N, Beeler J, test WHOwgomprn. Plaque reduction neutralization test for measles antibodies: Description of a standardised laboratory method for use in immunogenicity studies of aerosol vaccination. *Vaccine* 2007; **26**(1): 59-66.

16. Cohen BJ, Doblas D, Andrews N. Comparison of plaque reduction neutralisation test (PRNT) and measles virus-specific IgG ELISA for assessing immunogenicity of measles vaccination. *Vaccine* 2008; **26**(50): 6392-7.

17. Coughlin MM, Beck AS, Bankamp B, Rota PA. Perspective on Global Measles Epidemiology and Control and the Role of Novel Vaccination Strategies. *Viruses* 2017; **9**(1).

18. Immunological Basis for Immunization Series: Measles - Update 2009. Geneva, Switzerland, 2009.

19. Cho HK, Lee H, Kim HW, et al. Seroprevalences of Specific IgG Antibodies to Measles, Mumps, and Rubella in Korean Infants. *J Korean Med Sci* 2016; **31**(12): 1957-62.

20. Cohen BJ, Parry RP, Doblas D, et al. Measles immunity testing: comparison of two measles IgG ELISAs with plaque reduction neutralisation assay. *J Virol Methods* 2006; **131**(2): 209-12.

21. Hatchette TF, Scholz H, Bolotin S, et al. Calibration and Evaluation of Quantitative Antibody Titers for Measles Virus by Using the BioPlex 2200. *Clin Vaccine Immunol* 2017; **24**(1).

22. Tischer A, Gassner M, Richard JL, Suter-Riniker F, Mankertz A, Heininger U. Vaccinated students with negative enzyme immunoassay results show positive measles virus-specific antibody levels by immunofluorescence and plaque neutralisation tests. *J Clin Virol* 2007; **38**(3): 204-9.

23. Chapter 9. Manual for the laboratory-based surveillance of measles, rubella, and congenital rubella syndrome. Third ed: World Health Organization; 2018.

24. Whiting PF, Rutjes AW, Westwood ME, et al. QUADAS-2: a revised tool for the quality assessment of diagnostic accuracy studies. *Ann Intern Med* 2011; **155**(8): 529-36.

25. Leeflang MM, Deeks JJ, Gatsonis C, Bossuyt PM, Cochrane Diagnostic Test Accuracy Working G. Systematic reviews of diagnostic test accuracy. *Ann Intern Med* 2008; **149**(12): 889-97.

26. Shamseer L, Moher D, Clarke M, et al. Preferred reporting items for systematic review and meta-analysis protocols (PRISMA-P) 2015: elaboration and explanation. *BMJ* 2015; **350**: g7647.

27. van Enst WA, Ochodo E, Scholten RJ, Hooft L, Leeflang MM. Investigation of publication bias in meta-analyses of diagnostic test accuracy: a meta-epidemiological study. *BMC Med Res Methodol* 2014; **14**: 70.

28. The Cochrane Library. Cochrane screening and diagnostic tests methods group (SDTM). 2020. <https://methods.cochrane.org/sdt/welcome>. Accessed February 3, 2020.
